# Antibiotic class with potent in vivo activity targeting lipopolysaccharide synthesis in Gram-negative bacteria

**DOI:** 10.1073/pnas.2317274121

**Published:** 2024-04-05

**Authors:** Douglas L. Huseby, Sha Cao, Edouard Zamaratski, Sanjeewani Sooriyaarachchi, Shabbir Ahmad, Terese Bergfors, Laura Krasnova, Juris Pelss, Martins Ikaunieks, Einars Loza, Martins Katkevics, Olga Bobileva, Helena Cirule, Baiba Gukalova, Solveiga Grinberga, Maria Backlund, Ivailo Simoff, Anna T. Leber, Talía Berruga-Fernández, Dmitry Antonov, Vivekananda R. Konda, Stefan Lindström, Gustav Olanders, Peter Brandt, Pawel Baranczewski, Carina Vingsbo Lundberg, Edgars Liepinsh, Edgars Suna, T. Alwyn Jones, Sherry L. Mowbray, Diarmaid Hughes, Anders Karlén

**Affiliations:** ^a^Department of Medical Biochemistry and Microbiology, BMC, Uppsala University, Uppsala SE-75123, Sweden; ^b^Department of Medicinal Chemistry, BMC, Uppsala University, Uppsala SE-75123, Sweden; ^c^Department of Cell and Molecular Biology, BMC, Uppsala University, Uppsala SE-75123, Sweden; ^d^Latvian Institute of Organic Synthesis, Riga LV-1006, Latvia; ^e^Department of Pharmacy, Uppsala Drug Optimization and Pharmaceutical Profiling, Uppsala University, Uppsala SE-75123, Sweden; ^f^Department of Pharmacy, SciLifeLab Drug Discovery and Development Platform, Uppsala University, Uppsala SE-75123, Sweden; ^g^Bacteria, Parasites & Fungi, Statens Serum Institut, Copenhagen 2300, Denmark

**Keywords:** antibiotics, structure-based drug design, lipopolysaccharide, Gram-negative, LpxH

## Abstract

Here, we describe the identification of an antibiotic class acting via LpxH, a clinically unexploited target in lipopolysaccharide synthesis. The lipopolysaccharide synthesis pathway is essential in most Gram-negative bacteria and there is no analogous pathway in humans. Based on a series of phenotypic screens, we identified a hit targeting this pathway that had activity on efflux-defective strains of *Escherichia coli*. We recognized common structural elements between this hit and a previously published inhibitor, also with activity against efflux-deficient bacteria. With the help of X-ray structures, this information was used to design inhibitors with activity on efflux-proficient, wild-type strains. Optimization of properties such as solubility, metabolic stability and serum protein binding resulted in compounds having potent in vivo efficacy against bloodstream infections caused by the critical Gram-negative pathogens *E. coli* and *Klebsiella pneumoniae*. Other favorable properties of the series include a lack of pre-existing resistance in clinical isolates, and no loss of activity against strains expressing extended-spectrum-β-lactamase, metallo-β-lactamase, or carbapenemase-resistance genes. Further development of this class of antibiotics could make an important contribution to the ongoing struggle against antibiotic resistance.

## Significance

The ready availability of effective antibiotics underpins all of modern medicine. The ever-increasing spread of antimicrobial resistance continuously degrades the efficacy of existing antibiotics, thus requiring the development of innovative compounds and strategies simply to maintain the status quo. The lack of novel antibiotics to address this problem, particularly for difficult-to-treat Gram-negative infections, has been well documented. Here we describe the development of a unique antibiotic class targeting lipopolysaccharide synthesis, from an initial hit, to compounds with favorable drug-like properties and potent in vivo activity. This work verifies that the target of these compounds, LpxH in Gram-negative bacteria, is a viable target for antibiotics, and that the chemical series that we have investigated is promising for further development.

Antibiotics are an essential pillar of modern medicine. In many parts of the world, multidrug-resistant (MDR) and even pan-drug-resistant bacterial pathogens are becoming increasingly frequent and threaten to undermine the effectiveness of antibiotic therapy. In response to this threat, the World Health Organization (WHO), has issued a list of priority pathogens for which there is an urgent need to discover and develop new antibiotics (1). At the top of this list, classified as “critical priority,” are extended-spectrum beta-lactamase (ESBL) producing and carbapenem-resistant Gram-negative bacterial species: the Enterobacteriaceae, including *Escherichia coli* and *Klebsiella pneumoniae*, *Pseudomonas aeruginosa* and *Acinetobacter baumannii*. These species are designated as a critical priority for antibiotic development because they are increasingly resistant to the currently most effective classes of broad-spectrum antibiotics (3rd generation cephalosporins and carbapenems) and because they are responsible for a large proportion of community-acquired and nosocomial infections worldwide. Infections caused by antibiotic-resistant variants of these species are already having a huge negative impact on healthcare outcomes, both in low to middle-income and high-income countries (2, 3). Developing treatments for Gram-negative organisms is further complicated by the permeability differences between the outer and cytoplasmic membranes, which significantly limits the fraction of potential antibiotics that can access intracellular targets (4). Given the daunting scientific difficulties in developing new antibiotics against these organisms and the strong economic headwinds that the field has faced, it may not be surprising that no new class of Gram-negative-acting antibiotic has entered the market since fluoroquinolones in the 1970s. Efforts to develop new drugs have not stopped though and there exist promising new compounds under development, including the odilorhabdins, darobactin and ETX0462 (5–7).

**Fig. 1.**
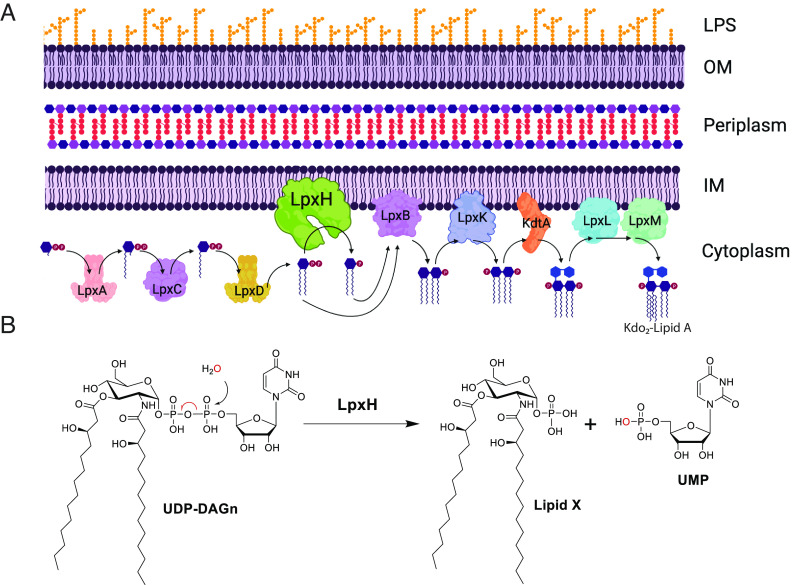
(*A*) Schematic representation of Gram-negative cell wall and the enzymes of the Raetz Kdo_2_-Lipid A biosynthetic pathway, highlighting the placement of LpxH in the pathway (13). For clarity, the UDP that is attached to the terminal phosphate for substrates up to the LpxH reaction is omitted from the figure. The product of the depicted reactions is Kdo_2_-Lipid A which is subsequently decorated with a core-oligosaccharide, flipped to the periplasmic-facing leaflet of the inner membrane, the O-antigen added, and ultimately directed to the outer leaflet of the outer membrane to form the LPS (12). (*B*) LpxH (UDP-2,3-diacylglucosamine hydrolase) catalyzes the fourth step in lipid A synthesis, hydrolyzing UDP-2,3-bis(3-hydroxymyristoyl) glucosamine to yield 2,3-bis(3-hydroxymyristoyl)-β-D-glucosaminyl 1-phosphate (lipid X) and uridine monophosphate.

Many years of research have shown that the bacterial cell wall is an excellent target for antibiotics (8, 9). The outer surface of the outer membrane (OM) of Gram-negative bacteria contains a unique structural component, lipopolysaccharide (LPS). LPS is partially responsible for OM structural integrity, maintaining a permeability barrier against hydrophobic and large hydrophilic compounds, including many antibiotics. Lipid A (PubChem CID 9877306), the hydrophobic portion of LPS, anchors it in the OM (10–12). The importance of LPS, and lipid A in particular, for the viability and virulence of Gram-negative bacteria (13) makes the inhibition of lipid A biosynthesis a promising target for the development of antibiotics (14, 15). Accordingly, antibiotics inhibiting lipid A biosynthesis should be widely effective against many Gram-negative pathogens. In *E. coli*, nine enzymes are required for the biosynthesis of lipid A (Fig. 1) and several different compound classes have been identified that inhibit the reactions catalyzed by the LpxA (acyl-[acyl-carrier-protein]-UDP-*N*-acetylglucosamine *O*-acyltransferase), LpxC (UDP-3- *O*-acyl-*N*-acetylglucosamine deacetylase), or LpxH (UDP-2,3-diacylglucosamine diphosphatase) enzymes in the pathway (14–17). The lipid A pathway has been further validated as an antibiotic target with the identification of ACHN-975, an inhibitor of LpxC that entered Phase I clinical trials, although these trials were stopped because of issues with cardiotoxicity (hypotension) and inflammation at the site of injection (18, 19). Development is continuing in this area with a promising new compound targeting LpxC showing oral availability and efficacy in mouse models being recently reported (20).

**Fig. 2.**
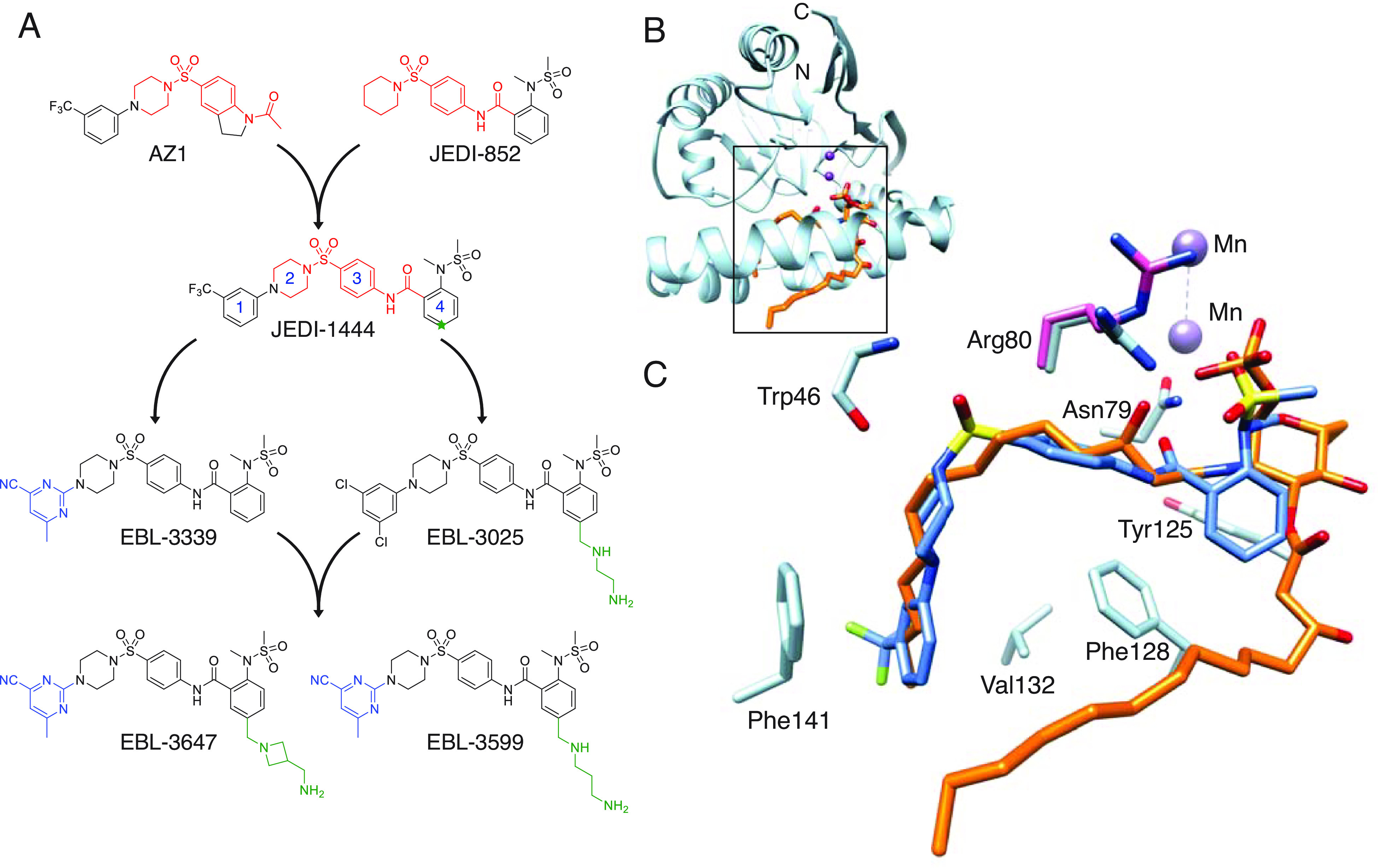
(*A*) Chemical optimization cascade for LpxH inhibitors. The shared structural features between AZ1, JEDI-852, and JEDI-1444 are marked in red. Numbers on JEDI-1444 indicate ring designations and C5 of ring 4 is indicated with a green star. (*B*) X-ray structure of *E. coli* LpxH in complex with lipid X (orange, PDB 8QJZ) at 1.35-Å resolution. Manganese ions (purple) roughly indicate the catalytic region of the enzyme. N- and C- termini of protein are labeled. (*C*) Detailed view of JEDI-1444 (in blue, PDB 8QK9) binding site from the complex with *E. coli* LpxH overlaid with the lipid X molecule (in orange) from the *E. coli* LpxH complex. Key amino acids interacting with JEDI-1444 are colored light blue and labeled. The Arg80 sidechain in the enzyme-product complex is shown colored pink to highlight the conformational change in the sidechain induced by inhibitor binding.

The LpxH enzyme cleaves the pyrophosphate bond of UDP-2, 3-bis(3-hydroxytetradecanoyl)-glucosamine (UDP-DAGn) to yield lipid X (PubChem CID 123907) and UMP (Fig. 1) in the fourth step of lipid A biosynthesis. Roughly 70% of Gram-negative bacteria, including the WHO critical priority Gram-negative pathogens for R&D of new antibiotics, utilize this enzyme (13). The other 30% of Gram-negative bacteria use different enzymes, LpxG and LpxI, to perform the same function (21, 22); the species utilizing LpxG and LpxI (e.g., *Chlamydia*, *Rickettsia*) are not generally considered high-risk for antibiotic resistance. AstraZeneca reported a compound, AZ1 (Fig. 2*A*), targeting LpxH in this pathway based on WGS of resistant mutants; overexpression of LpxH also reduced the sensitivity of *E. coli* to the inhibitor (23). AZ1 only showed activity against strains defective in drug efflux. Subsequent efforts to develop novel LpxH inhibitors based on the AZ1 scaffold, and the X-ray structure of LpxH in complex with AZ1, have resulted in compounds with only modest antibacterial activity against wild-type *K. pneumoniae*, and no stand-alone activity against wild-type *E. coli* (15, 24–26).

## Results

### Origin of Program.

In a search for new drug targets and starting points for antibiotic discovery, we initiated a series of phenotypic screens, based on publicly available chemical libraries and information gathered from the literature, focusing on compounds for which there was some evidence of Gram-negative antibacterial activity. In our initial screens, we evaluated activity against *tolC*-inactivated mutants of *E. coli*. The TolC protein is the outer membrane channel protein that is required for activity of several of the most important efflux pumps in *E. coli*, and inactivation of TolC essentially abolishes efflux pump activity (27). From these phenotypic screens, we identified JEDI-852 (Fig. 2*A*) from the PubChem Library (https://pubchem.ncbi.nlm.nih.gov/bioassay/573) (28), which in our hands showed a MIC of 4 to 8 μg/mL against efflux-defective *E. coli,* and low cytotoxicity (IC_50_ > 32 μM) on HepG2 eukaryotic cells. We successfully isolated resistant mutants against this compound. Whole genome sequencing (WGS) of these mutants suggested that JEDI-852 targeted LpxH (*SI Appendix*, Table S1). We resynthesized the AZ1 compound and confirmed the involvement of LpxH by WGS of selected resistant clones (*SI Appendix*, Table S1) (24–26). Both our own, and previously published mutations, map to the lipid X (enzymatic reaction product) binding site of LpxH (*SI Appendix*, Table S1 and Fig. S1*A*) (23). While the activity of JEDI-852 against efflux-defective strains was considered promising, the permeability and efflux properties of Gram-negative bacteria present imposing and often insurmountable barriers to drug development, so obtaining compounds with activity against efflux-proficient cells was essential to the project. On this basis, we decided to initiate a chemistry program to develop LpxH inhibitors possessing whole-cell activity against wild-type Gram-negative bacteria.

### New LpxH Inhibitor with Activity against Wild-type Strains.

We recognized common structural elements of our hit compound JEDI-852 and AZ1 (Fig. 2*A*, indicated in red). We were able to combine the target-binding features of these two compounds into a single new molecule, JEDI-1444. We were pleased to find that JEDI-1444 possessed good activity against efflux-proficient (wild-type) laboratory strains (Fig. 2*A* and Table 1). Consistent with the presumed activity against the Gram-negative-specific enzyme LpxH, no antibacterial activity was observed against *Staphylococcus aureus* (ATCC 29213, MIC > 64 mg/L). Studies with efflux-defective *E. coli* indicated a low frequency of resistance for JEDI-1444, and whole-genome sequencing of the resulting mutant strains confirmed that LpxH, particularly the substrate-binding site, remained the only target for antibacterial activity (*SI Appendix*, Table S1 and Fig. S1).

**Table 1.** Summary of compound properties

Compounds	MIC (mg/L)				*E. coli* LpxH enzyme inhibition IC_50_ (nM) (*SI Appendix*, Figs. S13–S19)	In vitro safety		Absorbsion, distribution, excretion and metabolism (ADME)			Physiochemical properties	
	*E. coli* ATCC 25922		*K. pneumoniae* ATCC 13883			Hemolysis (%)	HepG2 viability IC_50_ (µM)	Stability human liver microsomes CL_int_ (µL/mg/min)	Stability mouse liver microsomes CL_int_ (µL/mg/min)	Fraction unbound in human plasma (%)	Thermodynamic solubility (µM)	clogD
	Wild-type	Δ*tolC* efflux mutant	Wild-type	In 50% human serum								
JEDI-852	>64	4	>64	nd	1300	0.03	>64	nd	nd	nd	nd	2.67
AZ1	>64	2	>64	nd	520	2.1	22.5	nd	nd	nd	nd	3.71
JEDI-1444	2	≤0.125	2	32	2.6	0.02	>64	209	126	0.1	0.210	4.34
EBL-3025	1	≤0.125	0.5	0.5	3.4	1.98	49	42.2	31.0	0.1	4.00	1.64
EBL-3339	1	≤0.125	0.5	2	1.5	0.05	29	551	625	0.54	1.20	2.50
EBL-3647	2	≤0.125	0.5	0.25	2.2	0.13	54	3.1	< 5	2.5	1090	0.44
EBL-3599	2	≤0.125	1	0.25	3.5	0.06	55	< 5	< 5	2.0	1530	0.47

### X-ray Structures.

To enable a structure-based drug design approach for the optimization of JEDI-1444, we produced LpxH proteins from *E. coli* (EcLpxH) and *K. pneumoniae* (KpLpxH) and solved the X-ray crystal structures of various enzyme-product and inhibitor complexes during the optimization of the chemical series (*SI Appendix*, Table S2). These structures show the same fold described originally for *Haemophilus influenzae* LpxH (HiLpxH) in complex with the enzymatic reaction product, lipid X (PDB 5K8K) (29). As for HiLpxH, the EcLpxH and KpLpxH proteins copurified with a bound molecule of lipid X in the substrate binding site but we were able to develop protocols to displace the product and form enzyme–inhibitor complexes. The 1.35-Å resolution EcLpxH complex, in particular, provides a detailed view of this portion of the substrate-binding site (Fig. 2 and *SI Appendix*, Fig. S1). In contrast to the highly mutated EcLpxH structure previously published, the lid of our structure is generally well-ordered (30). As described for the HiLpxH/lipid X complex (29), the phosphorylated alpha-D-glucosamine unit makes an intricate set of hydrogen-bonding interactions with the enzyme, while the pair of lipophilic aliphatic chains point away from the catalytic site which lies near the two manganese ions shown in Fig. 2 *B* and *C*. The glucosamine-1-P group of lipid X is locked in position by a number of interactions with enzyme side chains; notably, the guanidino groups of Arg80 and Arg157 (Fig. 2 and *SI Appendix*, Fig. S1) form salt links with the phosphate group of the product. One of the lipid X aliphatic tails extends toward the protein surface and is partially solvent-exposed, while the other is completely buried in the enzyme interior (Fig. 2*B*). The nonpolar side chains that interact with this buried aliphatic chain are conserved in EcLpxH and KpLpxH, producing a roughly 120° bend in the aliphatic chain.

The complex of EcLpxH with JEDI-1444 at 1.9 Å resolution (Fig. 2*C*) shows that a major part of the ligand, the N-arylpiperazine sulfonamide (rings 1, 2, and 3), and follows the path of the buried aliphatic tail of the product, with the bend at the central sulfonamide moiety mimicking the bend seen for the product’s aliphatic tail. The aromatic ring 1 packs against the phenyl ring of Phe141 on one face, forming classical π–π interactions, while hydrophobic interactions with the side chain of Val132 are seen on the other face. The piperazine (ring 2) adopts a chair conformation that is buried in a space whose surface is lined with hydrophobic side chains (*SI Appendix*, Fig. S2). One oxygen of the central sulfonamide moiety makes a hydrogen bond with the main-chain nitrogen of Trp46, and a close contact with the main-chain oxygen of Arg80. One face of the phenyl group (ring 3) packs loosely against buried side chains in the enzyme interior, notably Phe82, Phe128, and Met156. The side chain of Arg80 changes from the conformation seen in the product complex (Fig. 2*C* and *SI Appendix*, Figs. S1 and S2), enabling its guanidino group to stack directly on the other face of ring 3. The carbonyl oxygen in the linkage between rings 3 and 4 accepts a hydrogen bond from the side chain of Asn79, a residue that coordinates one of the active site metal ions (*SI Appendix*, Fig. S2). Ring 4 is aromatic, making ring-edge interactions with aromatic side chains of the protein (Tyr125, Phe128), and only approximately overlapping with the glucosamine in the product complex (Fig. 2*C* and *SI Appendix*, Fig. S2). The orthosubstituted phenyl group (ring 4) points toward the catalytic site, positioned so that an oxygen atom of the sulfonamide makes hydrogen-bonding interactions with NH1 of Arg80. The edge of ring 4 is solvent accessible, providing a useful site for the addition of polar or charged groups to increase solubility.

These findings enabled a structure-based drug design approach in our hit-to-lead optimization.

### Optimization of Drug-like Properties.

In spite of the promising antimicrobial activity of the JEDI-1444 LpxH inhibitor, this compound had several limitations, including low solubility, poor metabolic stability, and weak antibacterial activity in the presence of serum (Table 1). To find more drug-like analogs, hit expansion and structure–activity relationship studies were initiated. We began with attempts to replace the central sulfonamide moiety with carbonyl, sulfone, or sulfoxide. Carbonyl replacement was not tolerated, and sulfone and sulfoxide derivatives had lower target affinity. Replacements of the N-aryl piperazine (rings 1 and 2) moiety with N-aryl piperidine or even 3-alkyl substituted azetidine were tolerated, however, the resulting compounds did not exhibit MIC or ADME profiles superior to that of the original hit. Opening ring 2 resulted in loss of antibacterial activity. Phenyl ring 3 could be successfully changed to pyridine but not to 5-membered ring heterocycles, and its saturation to cyclohexyl led to complete loss of activity. We also attempted to introduce small substitutions (F, Cl, Me, OMe, etc.) on rings 2 and 3. It was quickly recognized that modifications in the core of the ligand were not tolerated, resulting in molecules with low target affinity and/or poor efflux/permeability properties. Such molecules showed no activity on wild-type strains.

The relatively high lipophilicity and aromaticity of JEDI-1444 was potentially responsible for its poor ADME properties. Therefore, our optimization strategy aimed for the preparation of analogs with reduced lipophilicity [quantified as calculated-logD (clogD)] as well as introduction/replacement with less aromatic substituents. For the reasons discussed above, most optimization would have to come from the ring 1 and 4 regions of the molecule.

Since the pocket accommodating ring 1 is very lipophilic, the introduction of polarity here while retaining good target affinity was a challenging task. Aliphatic and cyclic aliphatic replacements of this phenyl ring were made, with piperazine or piperidine moieties as ring 2. Many of these modifications were very successful with respect to improvement in MIC activity but did not help to improve solubility or MIC activity in the presence of serum. Optimization of the π–π stacking interactions between ring 1 and Phe141 led to a library of substituted heteroaromatic compounds. As expected, the introduction of polarity here was a trade-off between lipophilicity and target binding. Nevertheless, to our satisfaction, many analogs showed significant improvements in serum MIC. The best compound in this group, EBL-3339, showed only minor reduction in MIC activity upon the addition of serum while retaining good target affinity.

At ring 4, we quickly found that moving the N-methylsulfonamide substituent from the ortho position was detrimental. This is in agreement with the crystallographic data showing the importance of hydrogen bonds between the sulfonyl oxygens and the side chain of Arg80. Varying the substituents on the nitrogen or sulfone components of this moiety, or the introduction of cyclic sulfonamides, did not deliver compounds with useful microbiological properties.

Guided by the X-ray structures, we designed a library of ligands bearing substituents on ring 4 that increase solubility. The X-ray structures revealed the possibility of using the binding region of the solvent-exposed acyl arm of lipid X (*SI Appendix*, Fig. S1) as a way to reach out to the solvent with polar substituents or cationic amines, which had the potential to improve compound accumulation in bacteria (31) The solvent-exposed environment around C5 of ring 4 (Fig. 2 *A* and *C*) can accommodate polar groups of various geometry and polarity allowing modulation of ADME properties without compromising target affinity.

By preparing and screening a diverse library of C5-modified ring 4 analogs we were able to identify a wide range of polar substituents that enhanced compound MIC profiles and, most importantly, dramatically improved activity in the presence of human serum. Unfortunately, the polar substituents tested gave only marginal increases in solubility, as exemplified by EBL-3025 (Table 1 and Fig. 2*A*).

Metabolite identification studies indicated that oxidative N-demethylation of the N-methylsulfonamide substituent on the ring 4 was the main metabolic event for the series. This process leads to an inactive des-methyl product. Since CYP450 binding sites are mostly lipophilic (32) we also expected that polar/charged C5 modifications on ring 4, being in the vicinity of the metabolic “soft spot,” might favorably affect stability. Indeed, most of the polar ring 4 C5 analogs showed prolonged half-life in microsomes, and all of the compounds bearing positive or negative charges at this position were metabolically very stable (Table 1).

The metabolic instability issue was now essentially solved, but the solubility remained suboptimal. At this point, it became apparent that changes only at one end of the molecule were not sufficient to overcome all of the ADME problems. Our next step was to combine the changes found to be useful on ring 1 and ring 4. A focused library of compounds featuring various combinations of the best ring 1 and ring 4 modifications was therefore made and tested. This led to the identification of EBL-3647 and EBL-3599 (Fig. 2*A*).

### Characterization of EBL-3647 and EBL-3599.

**Fig. 3.**
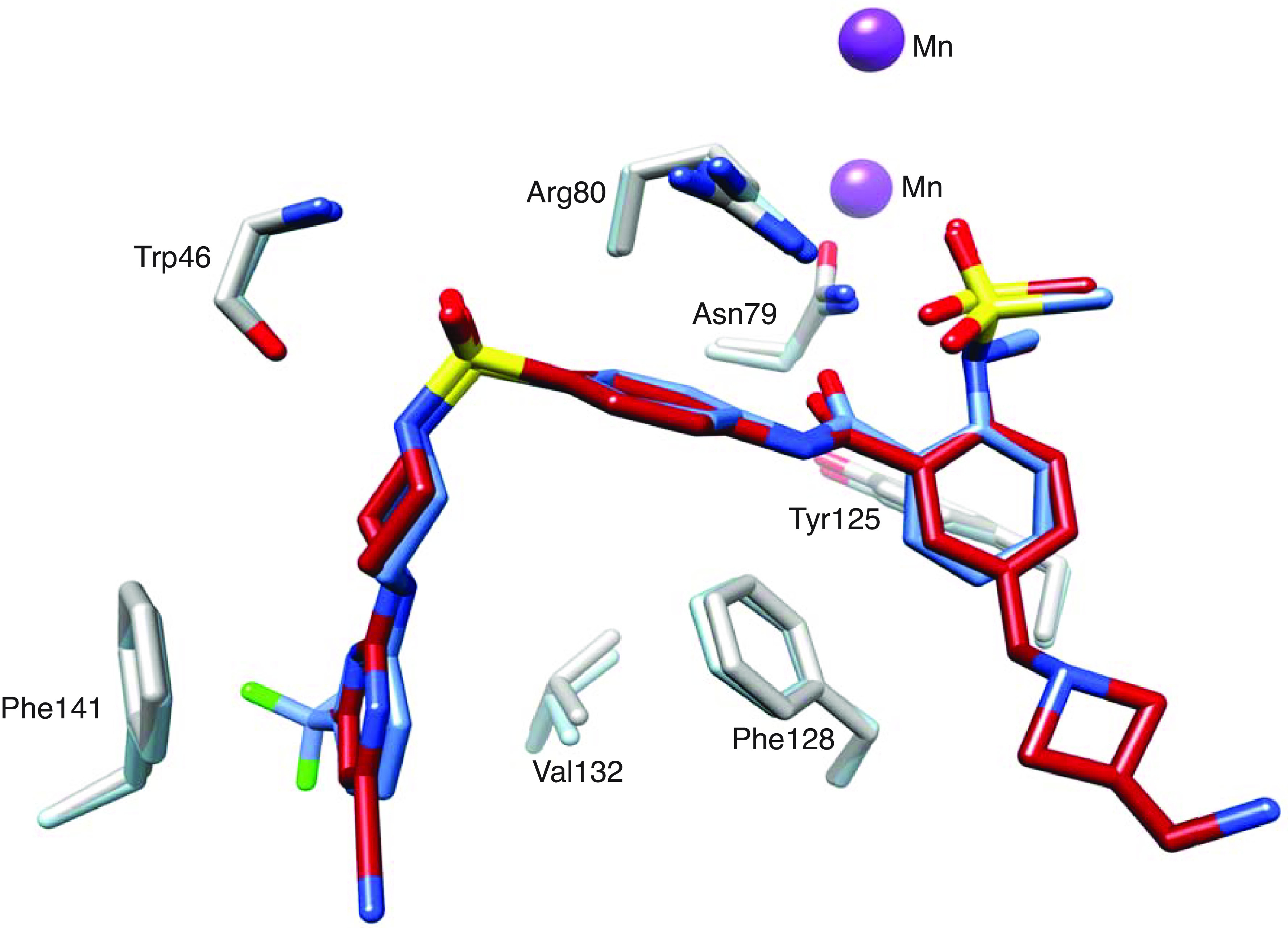
Views from X-ray structures of *E. coli* LpxH complex with JEDI-1444 (blue, PDB 8QK9) and *K. pneumoniae* LpxH complex with EBL-3647 (red, PDB 8QK5) overlaid. Amino acids from both the *E. coli* (steel blue) and *K. pneumoniae* (gray) crystal structures are shown to highlight the identical binding mode of the two compounds. The ring 4 C5 extension of EBL-3647 forms no specific interactions and electron density for the tip is poorly defined in the crystal structure.

Combining the ring 1 and 4 modifications yielded compounds that had lipophilicity (clogD) values improved by four to five log units compared to JEDI-1444. These modifications also resulted in improved MIC activity in the presence of serum, >5000-fold improvements in thermodynamic solubility, and >25-fold increase in stability in human- and mouse-liver microsomes (Table 1). The X-ray structure of the complex of EBL-3647 with KpLpxH shows that it retains the binding mode of the original hit compound JEDI-1444 (as well as EBL-3339), with both protein and ligand closely superimposable (Fig. 3 and *SI Appendix*, Figs. S2 and S3). As intended, the C5 substitution on ring 4 extends into the solvent, making no meaningful interactions with the protein. Both EBL-3647 and EBL-3599 had potent antimicrobial activity against wild-type *E. coli* and *K. pneumoniae* (Table 1), as well as having measurable MICs against efflux-defective *A. baumannii*, *P. aeruginosa,* and *P. mirabilis* (*SI Appendix*, Table S3). The activity of both compounds was unaffected by the presence of a diverse range of problematic resistance genes, including ESBL, MBL, and 16S ribosome-methyl-transferases (RMT), and their activity was maintained in carbapenem- and colistin-resistant isolates (*SI Appendix*, Table S4). They also possess low frequencies of resistance (*SI Appendix*, Tables S5–S7), low MICs, and unimodal MIC_90_s against clinical *E. coli* (4 mg/L for both compounds) and *K. pneumoniae* (8 mg/L for both compounds) isolates, similar to the MICs of laboratory strains (*SI Appendix*, Figs. S4 and S5). Like JEDI-1444, these compounds remain good substrates for efflux (Table 1), but their potent on-enzyme activity is sufficient to partially overcome this liability.

**Fig. 4.**
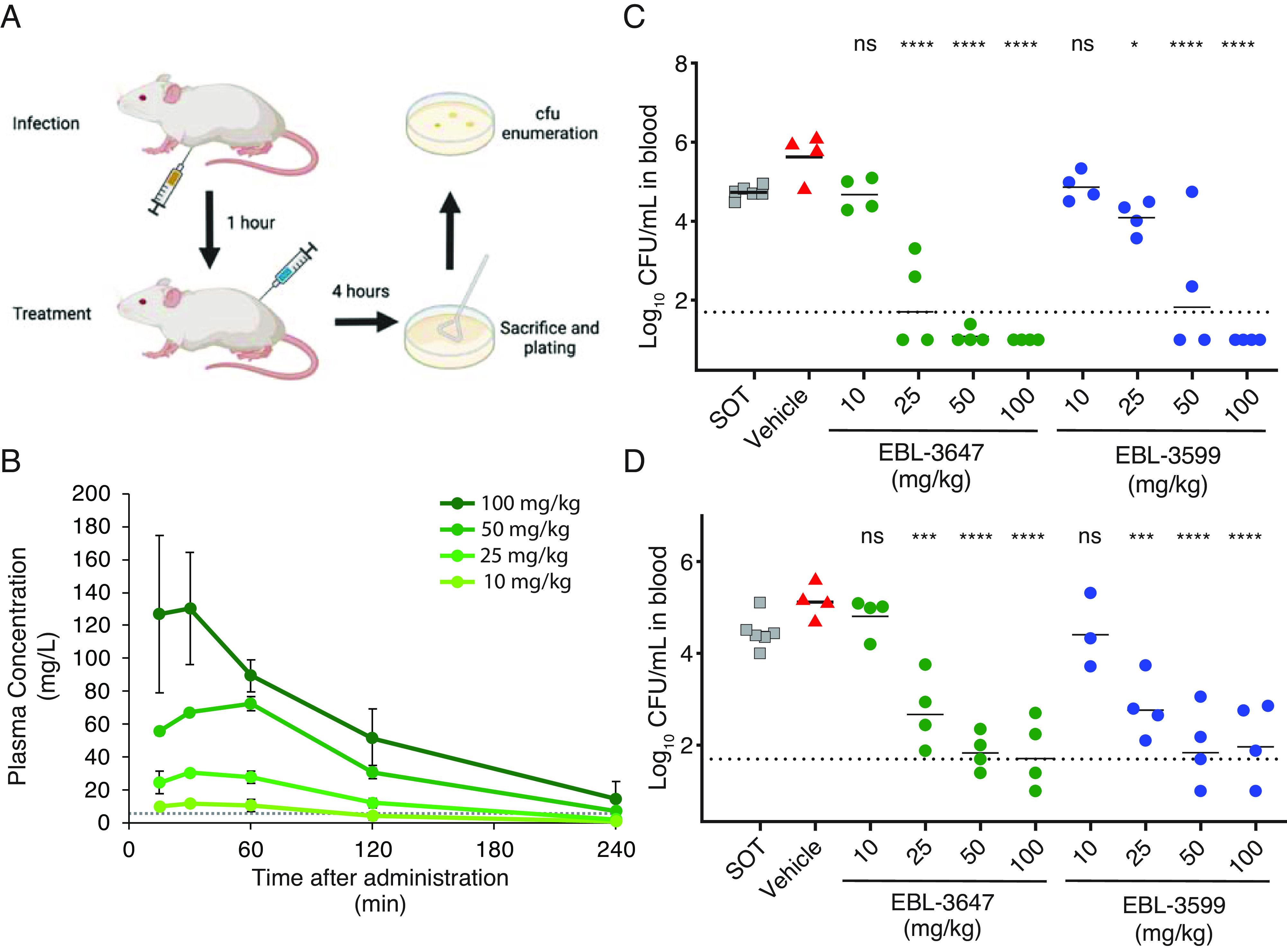
(*A*) Schematic of infection and treatment for in vivo peritoneal infection efficacy experiments. (*B*) Representative plasma PK graph for a single subcutaneous administration of EBL-3647 at various doses. The dotted line represents the MICs of the *E. coli* and *K. pneumoniae* strains used in the in vivo efficacy studies (4 mg/L). Results are represented as average concentrations ± SD from three to six mice. Corresponding data for EBL-3599 and calculated PK parameters can be found in *SI Appendix*, Fig. S6 and Tables S10 and S11. *E. coli* (EN122) (*C*) and *K. pneumoniae* (EN124) (*D*) blood bacterial counts in a dose–response efficacy study of EBL-3647 and EBL-3599 in a peritoneal infection model. The dotted line represents the limit of detection. Data for peritoneal fluid CFU can be found in *SI Appendix*, Figs. S7 and S8. Statistics for in vivo efficacy studies were calculated using a Dunnett’s multiple comparisons test versus vehicle treatment: ns not significant, *P < 0.1, ***P < 0.001, ****P < 0.0001.

Based on the excellent in vitro properties of these compounds, EBL-3647 and EBL-3599 were tested for in vivo efficacy. Both compounds were well tolerated with neither inducing adverse reactions in mice after intravenous administration at a dose of 50 mg/kg and subcutaneous administration at a dose of 100 mg/kg. Pharmacokinetic (PK) studies in mice showed that a range of single doses yielded plasma concentrations of compounds greater than the MIC for several hours (Fig. 4*B* and *SI Appendix*, Fig. S6). The compounds were then tested in dose–response studies against both *E. coli* and *K. pneumoniae* in a mouse peritonitis model (Fig. 4 and *SI Appendix*, Figs. S7 and S8). Doses of EBL-3647 and EBL-3599 ≥50 mg/kg yielded efficacy similar to ciprofloxacin (13 mg/kg) against *K. pneumoniae* (*SI Appendix*, Figs. S9 and S10). Estimation of efficacy parameters indicated ED_50_ for single doses in blood of 12.3 mg/kg (*E. coli*) and 21.0 mg/kg (*K. pneumoniae*) for EBL-3647, and 20.0 mg/kg (*E. coli*), and 20.2 mg/kg (*K. pneumoniae*) for EBL-3599 (*SI Appendix*, Figs. S11 and S12 and Tables S8 and S9).

## Discussion

Given the economic realities of antibiotics, it is not surprising that the vast majority of drugs currently in the development pipeline are modifications of existing drug scaffolds (33). Inventing new versions of existing drugs can circumvent project-threatening pitfalls that are extremely common in antibiotic development, including permeability-efflux problems in Gram-negative organisms, toxicity at the high doses at which antibiotics are typically administered, and unforeseen lack of efficacy in clinical trials (34). However, a dwindling pool of effective antibiotics for many infections due to inexorably increasing resistance means that new targets and strategies will soon be required (35).

There has been considerable interest in developing antibiotics that target the lipid A synthesis pathway since it is present in all of the WHO-designated critical priority pathogens for R&D of new antibiotics. Here, we succeeded in identifying a hit compound, JEDI-852, with good affinity to the LpxH enzyme in this pathway. Combining certain structural features of this compound with those of the only other LpxH inhibitor known at the time, AZ1, gave us a new scaffold with a vastly improved set of properties. Very early in the development, we were able to generate analogs with very high affinity to the target and good MICs, but their lipophilicity manifested in poor solubility, which combined with metabolic instability, made them suboptimal for use in vivo. In addition, protein binding resulted in a dramatic loss of MIC activity in the presence of serum. Fixing these ADME properties without sacrificing target affinity became a major challenge for further optimization of this series. The fact that our molecules bind in the very lipophilic part of the substrate-binding domain made this task especially difficult. Nevertheless, by substituting the phenyl ring 1 with polar heterocycles, and by introducing cationic extensions at C5 in ring 4, we managed to reduce clogD for advanced analogs by 4 to 5 log units with essentially no change in the enzymatic IC_50_. This progress allowed us to decrease protein binding and, as a result, to improve MIC activity in the presence of serum from 32 to 64 to 0.25 µg/mL. Fortuituously, the same C5 modifications at ring 4 that helped us to improve solubility also became an effective tool for fixing the metabolic stability problem. The net result of these improvements was compounds with favorable PK properties suitable for in vivo use.

A successful merging experiment, combining two good inhibitors to produce a better inhibitor, works best if the different binding sites are independent and do not force conformational changes in the new compound or in the binding site. Our studies show only small changes in the conformation of our inhibitors (*SI Appendix*, Fig. S3) such that the common atoms of JEDI-1444, JEDI-852, and AZ1 (PDB code 6PIB) (15) in their respective complexes have rmsd in the range 0.3 to 0.7 Å. The side chains with which they interact are highly conserved in sequence in EcLpxH and KpLpxH (*SI Appendix*, Fig. S2) which may explain why the in vitro activity of our compounds against additional species, including the WHO critical target pathogens *A. baumannii* and *P. aeruginosa,* is disappointing; in vitro activity has thus far only been observed in efflux-defective strains. These species are noted for being difficult to treat, and the possibility to further develop the compound series to obtain wild-type activity against these species is enticing. The resistant *E. coli* strains that we have produced highlight the importance of three residues in particular, Phe141, Phe128, Arg80 (*SI Appendix*, Tables S1 and S7), that play critical roles in forming the binding site for rings 1, 3, and 4, respectively. Residue 141 is a leucine in PaLpxH, while 128 is a leucine in both AbLpxH and PaLpxH. Indeed, the binding of the lipid X product to EcLpxH and PaLpxH structures shows differences at the ring 1-binding site (*SI Appendix*, Fig. S1*C*) that would be expected to affect inhibitor binding. EBL-3599 and EBL-3647 both show more than a factor of 10 decrease in inhibitory effect on PaLpxH compared to EcLpxH (*SI Appendix*, Table S3). This opens the possibility that efflux is not the only issue and that further development of this series could lead to compounds with broad-spectrum activity against all of the WHO critical target pathogens.

The LpxH inhibitors we have described here, EBL-3599 and EBL-3647, have potent in vitro activity against a diverse range of clinical *E. coli* and *K. pneumoniae* isolates, irrespective of the resistance genotype (*SI Appendix*, Table S4). The screened strains included isolates with extremely difficult-to-treat resistance profiles including ESBL, MBL, carbapenemase, and RMT producers, as well as phenotypically colistin-resistant strains including mcr-1 producing strains. The low, unimodal MIC_90_ values demonstrate that there is no detectable pre-existing resistance to this class of compounds (*SI Appendix*, Figs. S4 and S5). In spite of being excellent substrates for efflux pumps as demonstrated by the extremely low MIC values measured for efflux-defective *tolC* mutants (Table 1), we were rarely able to identify efflux mutations contributing to resistance in our frequency of resistance experiments (*SI Appendix*, Tables S1 and S7). We suspect that this indicates that these compounds are also excellent *inducers* of efflux pumps and that the bacteria find little benefit in further upregulation of pumps by mutation of negative regulators of efflux (*marR*, *ramR*, etc.). We demonstrate that these compounds targeting LpxH are also active against *E. coli and K. pneumoniae* in an in vivo peritonitis model. During the course of this infection model, bacteria spread to the bloodstream of the mice. The ability of these compounds to strongly reduce the number of bacteria recovered from blood in only a single-dose treatment highlights their potential to treat the most life-threatening infections with Gram-negative MDR pathogens. This confirms that this step in LPS biosynthesis, in addition to the previously explored LpxC, is a viable target for antibiotic development.

## Materials and Methods

### Chemistry.

All chemicals and solvents were purchased from Sigma Aldrich, Fisher Scientific, FluoroChem, Enamine and Ark Pharm chemicals. Intermediates have been characterized by^1^H NMR and ESI (MS) and all final compounds have been characterized by ^1^H NMR and ^13^C NMR and HRMS using Waters UPLC mass spectrometer with an ESI source. ^1^H and ^13^C NMR spectra were recorded on Varian Mercury Plus instrument; ^1^H at 399.9 MHz and ^13^C at 100.6 MHz at 25 °C. All final compounds were purified by RP HPLC and were ≥95% pure as determined by analytical HPLC and ^1^H NMR. Preparative RP-HPLC was performed on Gilson system equipped with Nucleodur C18 HTec column and analytical RP-HPLC was performed on a Gilson RP-HPLC system with Onyx Monolithic C18 column using UV detection at 220 nm and 254 nm. For in vitro testing, all the compounds were prepared as 10 mM DMSO stocks. Complete synthetic details available in *SI Appendix*.

### Biochemistry.

*E. coli*-optimized EcLpxH and KpLpxH sequences, cloned into a pET-26b(+) vector (Novagene) using NdeI/XhoI restriction sites, were ordered from GenScript Biotech (the Netherlands), giving constructs with a His_6_-tag at the C-terminal end, and bearing kanamycin resistance. Competent *E. coli* BL21-AI cells (Invitrogen) were transformed and grown at 37 °C in Luria broth containing 50 μg/mL kanamycin to an OD_600_ of 0.5 to 1.0, then induced for 2 to 3 h by adding IPTG and L-arabinose to final concentrations of 0.5 mM and of 2 mg/mL, respectively. Harvested cells were lysed in a One-Shot cell disrupter (Constant Systems Ltd., UK) in 20 mM Tris-HCl pH 8.0, 300 mM NaCl, 20 mM imidazole, 4 M urea, 5% (v/v) glycerol, 0.01 mg/mL RNase A and 0.02 mg/mL DNase, plus cOmplete, EDTA-free protease inhibitor cocktail (Roche). Proteins were purified using Ni-nitrilotriacetic acid agarose (Qiagen), HiTrap Heparin HP affinity chromatography (Cytiva), and HiLoad 16/60 Superdex 200 chromatography (Amersham Biosciences). Final samples were concentrated to 15 mg/mL protein and stored in aliquots in 20 mM Tris-HCl (pH 8.0) containing 300 mM NaCl and 2 mM DTT at −80 °C. Assays were performed essentially as described by ref. 26. Additional assay details are available in *SI Appendix*.

### Structural Biology.

Crystals were obtained overnight at ambient temperature from various conditions in Morpheus III screens (Molecular Dimensions) and cryocooled in the respective mother liquor. Diffraction data were collected at 100 K at the European Synchrotron Radiation Facility (ESRF, Grenoble) and Diamond Light Source (UK). Data processing, structure solution by molecular replacement, and refinement were carried out as described in *SI Appendix*.

### Microbiology.

Minimum inhibitory concentration (MIC) values were determined according to the Clinical Laboratory Standards Institute guidelines. Frequency of resistance was determined by plating ~5 × 10^8^ bacterial cells on agar-solidified media plates containing test compound at concentrations corresponding to four times the measured solid-media MIC of the strains. Colonies were counted after 24 h, restreaked onto media containing the same concentration of compound, and the MIC remeasured against the assayed compound. Genomic DNA from isolated clones was sequenced by paired-end sequencing on an Illumina MiSeq device. Complete experimental details available in *SI Appendix*.

### In Vivo Assays.

PK and safety studies were done the Latvian Institute for Organic Synthesis (Riga, Latvia) and in vivo efficacy studies were done at Statens Serum Institut (Copenhagen, Denmark). Assays were done in accordance with the relevant permits at the respective sites (*SI Appendix*). For PK studies, plasma samples were taken from mice dosed subcutaneously and the samples were analyzed by UPLC/MS/MS. For in vivo efficacy studies, neutropenic mice were infected intraperitoneally with 10^6^ bacteria and treated with subcutaneously with test compounds 1 h after infection. The mice were euthanized 4 h after treatment and serially diluted samples of blood and peritoneal fluid were plated onto blood agar plates. Complete experimental details available in *SI Appendix*.

### ADME and Physiochemical Assays.

Thermodynamic solubility was assessed by dissolving 2 to 3 mg of test compound to equilibrium in 100 mM KPO4-buffer, pH 7.4. The metabolic stability assay was performed in the presence of human and mouse liver microsomes by measurement of test compounds disappearance over time (60 min). The plasma protein binding assay, including stability estimation, was performed using the RED devices (Thermo Fisher Scientific) in the presence of human and mouse plasma. All samples from the in vitro ADME studies were quantified by liquid chromatography-triple quadrupole mass spectrometry (LC–MS/MS). The assays were performed as previously described (36) and the details are available in *SI Appendix*. Cytotoxicity was assessed by incubation of test compounds in the presence of HepG2 cells for 72 h as previously described (37).

## Supplementary Material

Appendix 01 (PDF)

## Data Availability

Structure factor diffraction data and final coordinates have been deposited at the Protein Data Bank (pdb.org) with entry codes 8QJZ, 8QK9, 8QKA, 8QK2, and 8QK5 (38–42), for the EcLpxH/Lipid X complex, EcLpxH/JEDI-1444, KpLpxH/JEDI-852, KpLpxH/EBL-3339, and KpLpxH/EBL-3647 structures, respectively.

## References

[1] E. Tacconelli , Discovery, research, and development of new antibiotics: The WHO priority list of antibiotic-resistant bacteria and tuberculosis. Lancet Infect. Dis. **18**, 318–327 (2018). 29276051 10.1016/S1473-3099(17)30753-3

[2] M. J. Carvalho , Antibiotic resistance genes in the gut microbiota of mothers and linked neonates with or without sepsis from low- and middle-income countries. Nat. Microbiol. **7**, 1337–1347 (2022). 35927336 10.1038/s41564-022-01184-y PMC9417982

[3] A. Tabah , Epidemiology and outcomes of hospital-acquired bloodstream infections in intensive care unit patients: The EUROBACT-2 international cohort study. Intensive Care Med. **49**, 178–190 (2023). 36764959 10.1007/s00134-022-06944-2 PMC9916499

[4] P. D. Manrique, C. A. López, S. Gnanakaran, V. V. Rybenkov, H. I. Zgurskaya, New understanding of multidrug efflux and permeation in antibiotic resistance, persistence, and heteroresistance. Ann. N.Y. Acad. Sci. **1519**, 46–62 (2023). 36344198 10.1111/nyas.14921 PMC9839546

[5] Y. Imai , A new antibiotic selectively kills Gram-negative pathogens. Nature **576**, 459–464 (2019). 31747680 10.1038/s41586-019-1791-1 PMC7188312

[6] L. Pantel , Odilorhabdins, antibacterial agents that cause miscoding by binding at a new ribosomal site. Mol. Cell **70**, 83–94.e7 (2018). 29625040 10.1016/j.molcel.2018.03.001

[7] T. F. Durand-Reville , Rational design of a new antibiotic class for drug-resistant infections. Nature **597**, 698–702 (2021). 34526714 10.1038/s41586-021-03899-0

[8] U. Theuretzbacher , Critical analysis of antibacterial agents in clinical development. Nat. Rev. Microbiol. **18**, 286–298 (2020). 32152509 10.1038/s41579-020-0340-0

[9] R. Tommasi, D. G. Brown, G. K. Walkup, J. I. Manchester, A. A. Miller, ESKAPEing the labyrinth of antibacterial discovery. Nat. Rev. Drug Discov. **14**, 529–542 (2015). 26139286 10.1038/nrd4572

[10] A. Troudi, J. M. Pagès, J. M. Brunel, Chemical highlights supporting the role of lipid A in efficient biological adaptation of gram-negative bacteria to external stresses. J. Med. Chem. **64**, 1816–1834 (2021). 33538159 10.1021/acs.jmedchem.0c02185

[11] J. C. Henderson , The power of asymmetry: Architecture and assembly of the gram-negative outer membrane lipid bilayer. Annu. Rev. Microbiol. **70**, 255–278 (2016). 27359214 10.1146/annurev-micro-102215-095308 PMC12914872

[12] B. Bertani, N. Ruiz, Function and biogenesis of lipopolysaccharides. EcoSal Plus **8** (2018). 10.1128/ecosalplus.esp-0001-2018 PMC6091223 30066669

[13] C. R. H. Raetz, C. M. Reynolds, M. S. Trent, R. E. Bishop, Lipid A modification systems in gram-negative bacteria. Annu. Rev. Biochem. **76**, 295–329 (2007). 17362200 10.1146/annurev.biochem.76.010307.145803 PMC2569861

[14] Z. Niu , Small molecule LpxC inhibitors against gram-negative bacteria: Advances and future perspectives. Eur. J. Med. Chem. **253**, 115326 (2023). 37023679 10.1016/j.ejmech.2023.115326

[15] J. Cho , Structural basis of the UDP-diacylglucosamine pyrophosphohydrolase LpxH inhibition by sulfonyl piperazine antibiotics. Proc. Natl. Acad. Sci. U.S.A. **117**, 4109–4116 (2020). 32041866 10.1073/pnas.1912876117 PMC7049123

[16] P. Zhou, J. Hong, Structure- and ligand-dynamics-based design of novel antibiotics targeting lipid A enzymes LpxC and LpxH in gram-negative bacteria. Acc. Chem. Res. **54**, 1623–1634 (2021). 33720682 10.1021/acs.accounts.0c00880 PMC9593327

[17] M. D. Ryan , Discovery of novel UDP-*N*-acetylglucosamine acyltransferase (LpxA) inhibitors with activity against *Pseudomonas aeruginosa*. J. Med. Chem. **64**, 14377–14425 (2021). 34569791 10.1021/acs.jmedchem.1c00888

[18] F. Cohen , Optimization of LpxC inhibitors for antibacterial activity and cardiovascular safety. ChemMedChem **14**, 1560–1572 (2019). 31283109 10.1002/cmdc.201900287

[19] K. M. Krause , Potent LpxC inhibitors with in vitro activity against multidrug-resistant Pseudomonas aeruginosa. Antimicrob. Agents Chemother. **63**, e00977-19 (2019). 31451507 10.1128/AAC.00977-19 PMC6811409

[20] J. Zhao , Preclinical safety and efficacy characterization of an LpxC inhibitor against Gram-negative pathogens. Sci. Transl. Med. **15**, eadf5668 (2023). 37556556 10.1126/scitranslmed.adf5668 PMC10785772

[21] L. E. Metzger, C. R. H. Raetz, An alternative route for UDP-diacylglucosamine hydrolysis in bacterial lipid A biosynthesis. Biochemistry **49**, 6715–6726 (2010). 20608695 10.1021/bi1008744 PMC2914816

[22] H. E. Young , Discovery of the elusive UDP-diacylglucosamine hydrolase in the lipid A biosynthetic pathway in Chlamydia trachomatis. mBio **7**, e00090 (2016). 27006461 10.1128/mBio.00090-16 PMC4807358

[23] A. S. Nayar , Novel antibacterial targets and compounds revealed by a high-throughput cell wall reporter assay. J. Bacteriol. **197**, 1726–1734 (2015). 25733621 10.1128/JB.02552-14 PMC4402386

[24] S. Kwak , Development of LpxH inhibitors chelating the active site dimanganese metal cluster of LpxH. ChemMedChem **18**, e202300023 (2023). 37014664 10.1002/cmdc.202300023 PMC10239344

[25] S.-H. Kwak , Synthesis and evaluation of sulfonyl piperazine LpxH inhibitors. Bioorg. Chem. **102**, 104055 (2020). 32663666 10.1016/j.bioorg.2020.104055 PMC7484203

[26] M. Lee , Structure-activity relationship of sulfonyl piperazine LpxH inhibitors analyzed by an LpxE-coupled malachite green assay. ACS Infect. Dis. **5**, 641–651 (2019). 30721024 10.1021/acsinfecdis.8b00364 PMC6730544

[27] J. Anes, M. P. McCusker, S. Fanning, M. Martins, The ins and outs of RND efflux pumps in Escherichia coli. Front. Microbiol. **6**, 587 (2015). 26113845 10.3389/fmicb.2015.00587 PMC4462101

[28] E. E. Bolton, Y. Wang, P. A. Thiessen, S. H. Bryant, “PubChem: Integrated platform of small molecules and biological activities” in Annual Reports in Computational Chemistry, R. A. Wheeler, D. C. Spellmeyer, Eds. (Elsevier, 2008), pp. 217–241.

[29] J. Cho, C.-J. Lee, J. Zhao, H. E. Young, P. Zhou, Structure of the essential Haemophilus influenzae UDP-diacylglucosamine pyrophosphohydrolase LpxH in lipid A biosynthesis. Nat. Microbiol. **1**, 16154 (2016). 27780190 10.1038/nmicrobiol.2016.154 PMC5081216

[30] H. O. Bohl , The substrate-binding cap of the UDP-diacylglucosamine pyrophosphatase LpxH is highly flexible, enabling facile substrate binding and product release. J. Biol. Chem. **293**, 7969–7981 (2018). 29626094 10.1074/jbc.RA118.002503 PMC5971466

[31] M. F. Richter , Predictive compound accumulation rules yield a broad-spectrum antibiotic. Nature **545**, 299–304 (2017). 28489819 10.1038/nature22308 PMC5737020

[32] D. A. Smith, K. Brown, M. G. Neale, Chromone-2-carboxylic acids: Roles of acidity and lipophilicity in drug disposition. Drug. Metab. Rev. **16**, 365–388 (1985). 3915471 10.3109/03602538508991440

[33] C. Årdal , Antibiotic development—Economic, regulatory and societal challenges. Nat. Rev. Microbiol. **18**, 267–274 (2020). 31745330 10.1038/s41579-019-0293-3

[34] N. K. Prasad, I. B. Seiple, R. T. Cirz, O. S. Rosenberg, Leaks in the pipeline: A failure analysis of gram-negative antibiotic development from 2010 to 2020. Antimicrob. Agents Chemother. **66**, e0005422 (2022). 35471042 10.1128/aac.00054-22 PMC9112940

[35] U. Theuretzbacher, K. Outterson, A. Engel, A. Karlén, The global preclinical antibacterial pipeline. Nat. Rev. Microbiol. **18**, 275–285 (2020). 31745331 10.1038/s41579-019-0288-0 PMC7223541

[36] A. E. Cotman , Discovery and hit-to-lead optimization of benzothiazole scaffold-based DNA gyrase inhibitors with potent activity against *Acinetobacter baumannii* and *Pseudomonas aeruginosa*. J. Med. Chem. **66**, 1380–1425 (2023). 36634346 10.1021/acs.jmedchem.2c01597 PMC9884090

[37] E. Lindhagen, P. Nygren, R. Larsson, The fluorometric microculture cytotoxicity assay. Nat. Protoc. **3**, 1364–1369 (2008). 18714304 10.1038/nprot.2008.114

[38] S. Sooriyaarachchi, T. Bergfors, T. A. Jones, S. L. Mowbray, Crystal structure of *E. coli* LpxH in complex with lipid X. PDB. https://www.rcsb.org/structure/8QJZ. Deposit 14 September 2023.

[39] S. Sooriyaarachchi, T. Bergfors, T. A. Jones, S. L. Mowbray, Structure of *E. coli* LpxH in complex with JEDI-1444. PDB. https://www.rcsb.org/structure/8QK9. Deposit 14 September 2023.

[40] S. Sooriyaarachchi, T. Bergfors, T. A. Jones, S. L. Mowbray, Structure of *K. pneumoniae* LpxH in complex with JEDI-852. PDB. https://www.rcsb.org/structure/8QKA. Deposit 14 September 2023.

[41] S. Sooriyaarachchi, T. Bergfors, T. A. Jones, S. L. Mowbray, Structure of *K.pneumoniae* LpxH in complex with EBL-3339. PDB. https://www.rcsb.org/structure/8QK2. Deposit 14 September 2023.

[42] S. Sooriyaarachchi, T. Bergfors, T. A. Jones, S. L. Mowbray, Structure of *K. pneumoniae* LpxH in complex with EBL-3647. PDB. https://www.rcsb.org/structure/8QK5. Deposit 14 September 2023.

