# Supplementary material for: Antibiotic class with potent in vivo activity targeting lipopolysaccharide synthesis in Gram-negative bacteria
Source: Proc Natl Acad Sci U S A. 2024 Apr 5;121(15):e2317274121. doi: 10.1073/pnas.2317274121 (PMC11009625; doi:10.1073/pnas.2317274121)
Supplement: Supplementary file 1 — Appendix 01 (PDF) [file pnas.2317274121.sapp.pdf]

PNAS

Supporting Information for

Antibiotic class with potent *in vivo* activity targeting lipopolysaccharide synthesis in Gram-negative bacteria

Douglas L. Huseby*, Sha Cao, Edouard Zamaratski, Sanjeewani Sooriyaarachchi, Shabbir Ahmad, Terese Bergfors, Laura Krasnova, Juris Pelss, Martins Ikaunieks, Einars Loza, Martins Katkevics, Olga Bobileva, Helena Cirule, Baiba Gukalova, Solveiga Grinberga, Maria Backlund, Ivailo Simoff, Anna T. Leber, Talía Berruga-Fernández, Dmitry Antonov, Vivekananda R. Konda, Stefan Lindström, Gustav Olanders, Peter Brandt, Pawel Baranczewski, Carina Vingsbo Lundberg, Edgars Liepinsh, Edgars Suna, T. Alwyn Jones, Sherry L. Mowbray , Diarmaid Hughes, Anders Karlén

*Corresponding author
Email: douglas.huseby@imbim.uu.se

This PDF file includes:

Supplementary Methods (Pages 2-20)
Supplementary Results (Pages 21-24)
Supplementary Figures S1-S19 (Pages 25-40)
Supplementary Tables S1-S11 (Pages 41-49)
Supplementary References (Pages 50-51)

Supplementary Methods

Biochemistry

Cloning, expression and purification of LpxHs. *E. coli*-optimized EcLpxH and KpLpxH sequences, cloned into a pET-26b(+) vector (Novagene) using NdeI/XhoI restriction sites, were ordered from GenScript Biotech (the Netherlands), giving constructs with a His_6-tag at the C-terminal end, and bearing kanamycin resistance. The correctness of the sequences was verified by DNA sequencing. Competent *E. coli* BL21-AI cells (Invitrogen) were transformed with the resulting plasmids for protein production.

Cultures of 8 L of Luria broth containing 50 µg/mL kanamycin were grown at 37 °C to an OD_{600} of 0.5-1.0, then induced by adding IPTG to a final concentration of 0.5 mM and L-arabinose to a final concentration of 2 mg/ml. After 2-3 h of incubation at 37 °C, cells were harvested by centrifugation, and the pellet was resuspended in lysis buffer (20 mM Tris-HCl pH 8.0, 300 mM NaCl, 20 mM imidazole, 4 M urea, 5% (v/v) glycerol, 0.01 mg/mL RNase A and 0.02 mg/mL DNase, plus one tablet of cOmplete, EDTA-free cocktail protease inhibitor cocktail (Roche)). Cells were lysed using a One-Shot cell disrupter (Constant Systems Ltd., UK). The lysate was spun down and the soluble fraction containing LpxH was incubated at 4 °C overnight with 1.5 mL pre-equilibrated Ni-nitrilotriacetic acid agarose (Qiagen) slurry using the same buffer. The column was then washed in steps of 30 column volumes with lysis buffer (without RNase A, DNase and protease inhibitor) containing 3, 2, 1, 0.5 and 0 M of urea. The protein was then eluted with eight column volumes of 500 mM imidazole in the same buffer. Fractions containing the desired protein as judged by SDS gels were pooled and purified on a HiTrap Heparin HP affinity column (Cytiva) (loading buffer 20 mM Tris-HCl, pH 8.0 and 5% glycerol, 2 mM DTT; elution buffer 20 mM Tris-HCl, pH 8.0 and 5% glycerol, 1 M NaCl, 2 mM DTT) and further applied to a HiLoad 16/60 Superdex 200 preparative grade column (Amersham Biosciences) pre-equilibrated with 20 mM Tris-HCl, pH 8.0, 300 mM NaCl, 5% glycerol, 2 mM DTT. The LpxH-containing fractions were concentrated using a Vivaspin concentrator (10 kDa cutoff, Vivascience). The final samples (up to 15 mg/ml protein, as estimated using molecular weights of 27542.68 and 27967.9 Da, and extinction coefficients of 28085 and 33585 M^{-1} cm^{-1} for EcLpxH2 and KpLpxH, respectively, calculated using the ProtParam tool(1) were analyzed by SDS PAGE and stored in 20 mM Tris-HCl (pH 8.0) containing 300 mM NaCl and 2 mM DTT at -80 °C.

Preparation of MBP-AaLpxE. A plasmid containing an N-terminal His_{10}-MBP-AaLpxE insert (pET26b-pelB-His-MBP-*lpxE$_{AA}$*(2), the kind gift of Prof Pei Zhou of Duke University) was transformed into *E. coli* C41(DE3) competent cells. The cells were grown to OD_{600} 0.4 in Luria broth containing 50 µg/ml kanamycin at 37 °C, and the expression was carried out for 3 hrs at 37 °C after induction with 1 mM IPTG. The fused MBP-AaLpxE protein was purified using TALON metal affinity resin, and after buffer exchange stored at -80 °C.

MBP-AaLpxE-coupled LpxH activity assay. The AaLpxE-coupled LpxH activity assay was based on a previously published protocol(3). Substrate concentration was calculated using a molecular weight of 1016 g/mol, and assuming that all absorbance at 262 nm is due to the UDP moiety, with an extinction coefficient of 9.9 $mM^{-1}cm^{-1}$. Briefly, the substrate mixture and the enzyme

mixture were prepared, each containing assay buffer (20 mM Tris-HCl at pH 8.0, 0.5 mg/ml BSA, 0.02% Triton X-100, 1 mM $MnCl_2$, 1 mM DTT, and 10% DMSO), and either 40 μM UDP-DAGn or 5 ng/ml LpxH (resulting in final concentrations of 20 μM and 0.1 nM, respectively, in the assay). The inhibitor was added to the enzyme mixture at the desired concentration. The two mixtures were pre-incubated at 37 °C for 10 min before starting the reaction by combining an equal amount of each mixture at 37 °C. Aliquots of 20 µl reaction mixture were removed at 30 min, and added to separate wells in a 96-well half-area plate containing 5 mM EDTA (final concentration) to quench the reaction after 30 minutes. MBP-AaLpxE was then added to a final concentration of 5 µg/ml in each well, and the samples incubated for 30 min at 37 °C. The LpxE reaction was then quenched by the addition of 3.75 M formic acid (final concentration) to the reaction mixture. In the following step, malachite green reagent (Sigma Aldrich) was added to a five-fold dilution, and the absorbance at 620 nm was measured using a plate reader (Envision, PerkinElmer) after incubation at room temperature for 30 min. The amount of free inorganic phosphate liberated during the LpxE reaction was calculated from a phosphate standard curve and used to determine the activity of the LpxH enzyme. The standard curve was prepared using the phosphate standard provided with the kit, which is diluted into the reaction buffer to provide a range of phosphate concentrations up to 40 µM (the linear range extends to 200 μM). Assays were run in triplicate.

The steady-state kinetic parameters, k_{cat} and K_m, were determined to be 70 ± 10 µM and 139 s^{-1}, respectively.

IC_{50} determinations. IC_{50}s of inhibitors were determined with assays as described above, using a series of inhibitor concentrations. The signal obtained from the reaction mix containing the inhibitor was compared to that of the uninhibited reaction to calculate relative enzyme activity. In the program Prism (Graph Pad Prism version 5.0d for Mac OSX, Graph Pad Software, San Diego California USA, www.graphpad.com), the activity was plotted against the log of the inhibitor concentration and fitted to the three-parameter equation (Eq. 1), where *Lo* is the estimated lowest enzyme activity at the highest inhibitor concentration, *Hi* is the highest enzyme activity for the uninhibited reaction, *Y* is the relative activity and *X* is the log of the inhibitor concentration. All experiments were run in triplicate. Curves and standard deviations are shown in the Supplementary Data.

$$Y = Lo + \frac{Hi-Lo}{(1+10^{(X-LogIC_{50})}} \text{ (Eq. 1)}$$

Structural Biology

Crystallization. For complex structures, inhibitors were added to diluted protein sample (protein:compound ratio 1:10) in the presence of 1 mM $MnCl_2$; the solution was then concentrated to ~15 mg/mL in 20 mM Tri-HCl, pH 8.0, 300 mM NaCl, 5% glycerol, 2 mM DTT, and 1 mM $MnCl_2$, prior to crystallization screening. Experiments were set up in 2-well MRC plates with a Mosquito crystallization robot (TTP Labtech). Drops consisting of 100 nL protein and 100 nL screening solution were equilibrated against 50 μl of the respective screening

solution. Two screens(4) were tested at ambient temperature; crystals appeared overnight. The EcLpxH/product crystals used for diffraction were harvested from Morpheus III condition G7 (1.2 % Cholic acid derivatives mix, 0.1 M Buffer System 2, pH 7.5, 50 % Precipitant Mix 3) and the EcLpxH/JEDI-1444 complex from Morpheus III condition G9 (1.2 % Cholic acid derivatives mix, 0.1 M Buffer System 3, pH 8.5, 50 % Precipitant Mix 1). The KpLpxH/JEDI-852 crystals were harvested from Morpheus III condition G12 (1.2 % Cholic acid derivatives mix, 0.1 M Buffer System 3, pH 8.5, 50 % Precipitant Mix 4) and both KpLpxH/EBL-3399 and KpLpxH-3647 crystals were harvested from Morpheus III condition G6 (1.2 % Cholic acid derivatives mix, 0.1 M Buffer System 2, pH 7.5, 50 % Precipitant Mix 2).

Data collection, structure solution, refinement, and model building. Crystals were harvested from the wells and cryo-cooled in the respective mother liquor. X-ray data were collected at 100 K at the European Synchrotron Radiation Facility (ESRF, Grenoble) and Diamond Light Source (UK), processed with XDS33(5) and scaled with AIMLESS34(6). Both EcLpxH complexes, as well as the KpLpxH/EBL-3647 complex, suffered from anisotropy in their diffraction patterns, as indicated. Statistics for X-ray data collection and model refinement are given in *SI Appendix*, Table S2.

Our first structure, the KpLpxH/Lipid X complex, was solved by molecular replacement with Phaser(7) using a search model derived from the highly mutated EcLpxH structure(5). Since this structure lacked all of the lid region, extensive rebuilding was required to model the complete lid. All subsequent structures were solved based on our own models using molecular replacement or crystallographic refinement as appropriate. Each model was improved by multiple cycles of restrained crystallographic refinement using REFMAC5(8) and rebuilt as needed in O(9). The occupancy of the carbonyl oxygens of residues 213-215 was set to zero at the start of each refinement, and solvents were added using the Water-tools in O, guided by the height of the peaks in the difference Fourier at the three carbonyl oxygens. Initial inhibitor models were built and their stereochemical restraints generated in O with the Qds-tools. The fit of the product or inhibitor to the final electron density is excellent in each complex, except in the rare instances where some missing atoms were noted. The overall fit of the main chain of each respective enzyme model is also excellent, with no significant breaks at the RMS of the map, except for one region that is disordered in all enzyme-inhibitor complexes, but well defined in the enzyme-product complex, as described in the Supplementary Material. The quantitative goodness of fit of our models has been analyzed with a new tool, the Maximum Enveloping Electron Density (MEED) score, also described in more detail in the Supplementary Information. Structural comparisons were made using the Lsq-tools in O with a close pair cut-off of 1 Å between CA atoms, unless explicitly started otherwise.

Final structures were deposited at the Protein Data Bank (pdb.org) with entry codes 8QJZ, 8QK9, 8QKA, 8QK2 and 8QK5, for the EcLpxH/Lipid X complex, EcLpxH/JEDI-1444, KpLpxH/JEDI-852, KpLpxH/EBL-3339 and KpLpxH/EBL-3647 structures, respectively.

In vitro ADME assays

Plasma protein binding and plasma stability in human and animal plasma. Pooled, anonymized human plasma was provided by Uppsala Academic Hospital and was collected from two donors (non-smoking) (citric acid). In brief, 0.2 ml of the plasma (50% plasma, 50% isotonic buffer) test solution (typically 10 µM final compound concentration) was transferred to the membrane tube in the RED insert (ThermoFisher Scientific). 0.35 ml isotonic phosphate buffer pH 7.4 was added to the other side of the membrane. The 96-well base plate was then sealed with an adhesive plastic film (Scotch Pad) to prevent evaporation. The sample was incubated with rapid rotation (900 rpm) on a Kisker rotational incubator at 37°C for 4 h to achieve equilibrium. A stability test of the test solution was prepared (to allow detection of drug degradation), 100 µL of the plasma test solution (in a plastic vial or on a sealed plate) was incubated at 37°C for 4 h (or as long as the dialysis time). The plasma test solution was frozen directly after the administration to prevent any degradation. Prior to LC-MS/MS analysis the plasma and buffer sample were treated with the addition of Methanol (1:3) containing Warfarin as internal standard to precipitate proteins. The standard curve was created using the plasma standard. The plate is then sealed, centrifuged and the supernatant is analysed by liquid chromatography coupled to triple quadrupole mass spectrometry (LC-MS/MS).

Metabolic stability in the presence of human and animal liver microsomes. Metabolic stability was determined in 0.5 mg/ml human or animal liver microsomes at a compound concentration of 1 µM in 100 mM KPO4 buffer pH 7.4 in a total incubation volume of 500 µl. The reaction was initiated by addition of 1 mM NADPH. At various incubation times, i.e. at 0, 5, 10, 20, 40 and 60 min, a sample was withdrawn from the incubation and the reaction was terminated by addition of cold acetonitrile with warfarin as an internal standard. The amount of parent compound remaining was analysed by liquid chromatography coupled to triple quadrupole mass spectrometry (LC-MS/MS).

Thermodynamic solubility. Thermodynamic solubility assay utilized solid form of a test compound. Solid test compound (~2-3 mg) was weighed in a glass HPLC vial and 100 mM KPO_4-buffer, pH 7.4 was added to give a theoretical max concentration if everything is dissolved of ~5-6 mg/ml. The vial was incubated at 900 rpm, 37 °C in a rotational shaker for 24h. After the incubation an aliquot (200 µl) was transferred to a glass insert and centrifuged at 10 000xg, 37 °C for 20 min to separate any solid material from the solution. The supernatant was transferred to a new HPLC vial and analysed by liquid chromatography coupled to triple quadrupole mass spectrometry (LC-MS/MS).

Liquid chromatography coupled to triple quadrupole mass spectrometry (LC-MS/MS). The test compounds were optimized on a Waters Acquity UPLC XEVO TQ-S micro system (Waters Corp.) operating in multiple reaction monitoring (MRM) mode with positive or negative electrospray ionization. Compounds were optimized by using the QuanOptimize software (Waters Corp.).

For chromatographic separation, a C18 BEH 1.7 µm column was used, with a general gradient of 1% to 90% of mobile phase B over a total running time of 2 min. Mobile phase A consisted of 5% acetonitrile and 0.1% formic acid in purified water, and mobile phase B of 0.1% formic acid in 100% acetonitrile. The flow rate was set to 0.5 ml/min and 5 µL of the sample was injected.

Cytotoxicity. The cytotoxicity assay was performed in HepG2 cells as previously described(10)

Microbiology

Minimum inhibitory concentration (MIC) determination. Broth microdilution and agar dilution MIC assays were conducted according to CLSI guidelines (M07-2012). Appropriate inoculation suspension density was assessed using a Sensititre Nephelometer with a Sensititre 0.5 McFarland Standard. Serum MICs were conducted in 50% heat-inactivated pooled human serum (Sigma-Aldrich). Serum was heated to 56˚C for one hour and then filtered using a 0.2 µm filter prior to use.

Hemolysis. Hemolysis was conducted as previously described (11).

Frequency of Resistance. Frequency of resistance was assessed by spread plating between 10^8 and 10^9 cfu of a freshly-grown culture of the strain of interest on plates containing a concentration of compound corresponding to 4 times the MIC of the strain (as determined by agar-dilution MIC). Serial dilutions of the same cultures were plated on non-selective media to determine the number of cells plated. The frequency of resistance was determined by dividing the number of colonies obtained on the selective plates after 24 hours of growth by the calculated total population plated. Typically 4 independently grown cultures were used to initiate 4 separate experiments, and the total combined number of obtained colonies and plated populations were used for calculations. Isolated colonies from selection plates were re-streaked on an additional plate containing the same concentration of compound used in the original selection. Clones were saved at -80˚C for subsequent analysis.

Whole-genome sequencing and data analysis. Genomic DNA of selected clones was prepared using an Epicentre MasterPure Complete DNA Purification Kit. Whole-genome sequencing libraries were prepared using an Illumina Nextera XT Library Preparation Kit. The libraries were sequenced using an Illumina MiSeq device with a V3 600-cycle reagent kit. Whole-genome sequencing data was analyzed using CLC Genomics Workbench Version 11. To determine the genotype of MDR strains from the ENABLE strain collection, *de novo* assembled contigs were analyzed using ResFinder (12–14)

Strain construction. The efflux-defective *P. mirabilis* strain EN1490 was constructed using EZ-Tn5 <KAN-2> Transposome mutagenesis with TypeOne Restriction Inhibitor. Approximately 3000 independent clones were screened for increased susceptibility to chloramphenicol (8 mg/L), yielding 2 independent clones with insertions in the gene *tolC*. One clone was chosen for screening the determine the contribution of efflux to the efficacy of these compounds in wild-type *P. mirabilis*.

In vivo pharmacokinetic studies

Animals. NMRI female mice (10–12 weeks old, 27–33 g, n = 60) were obtained from ENVIGO (The Netherlands) and housed prior to treatment under standard conditions (21–23 °C, 12-h light/dark cycle, relative humidity 45%–65%) with unlimited access to food (R70 diet from

Lantmännen) and water. The experimental procedures were performed in accordance with the guidelines of the European Community and local laws and policies (Directive 2010/63/EU), and all of the procedures were approved by Food and Veterinary Service, Riga, Latvia. Animals were weighed on the day of the treatment to calculate the required amount of compound for the corresponding dose.

Safety testing in mice. Mice were dosed intravenously or subcutaneously via a bolus injection at selected doses. Mice were extensively observed and toxicity signs were scored after 0-2, 5, 10, 15, 30, 60, 120, 240 min, and 24 h .

PK assay in mice. For PK studies mice were dosed subcutaneously (SC) via a single bolus injection (injection volume 10 mL/kg). Blood samples were collected in tubes containing heparin. Blood was sampled from tail vein 15, 30, 60, 120, and 240min after the SC administration of the compounds. Tubes were centrifuged at +4 °C 10,000× g for 3 min. Plasma samples were collected and stored at −20 °C until analysis.

Plasma sample analysis by UPLC/MS/MS. Concentrations of EBL-3599 and EBL-3647 in mouse blood plasma were measured by liquid chromatography tandem mass spectrometry (LC/MS/MS) in positive electrospray ionization (ESI+) in multi reaction monitoring (MRM) mode.

Analyses were performed on a Waters Acquity UPLC system coupled to Waters XEVO TQ-Smicro tandem mass spectrometer. Chromatographical separation was carried out on Waters Acquity BEH C18 column (2.1×50mm, 1.7µm). The column temperature was 30°C, flow rate was 0.4mL/min. Linear gradient elution was applied, solvent A was 0.1% formic acid in water and solvent B was acetonitrile. The gradient program was: 0min – 5% B; 2.5min – 98% B; 4.5min – 98% B; 4.7min – 5% B; 5min – 5% B. Injection volume was 1µL.

Mass spectrometry parameters were obtained experimentally by direct infusion of EBL-3599 and EBL-3647 standard solutions. MS/MS transitions for EBL-3599 were 656.00 > 200.00, 656.00 > 202.00, 656.00 > 219.00, 656.00 > 226.00 and 656.00 > 518.00. Cone voltage vas 15V and collision energy was 30 for all transitions. MS/MS transitions for EBL-3647 were 667.80 > 225.90 and 667.80 > 309.96. Cone voltage was 15V and collision energy was 35 and 25, respectively.

Calibration standards and quality control samples were prepared by spiking a blank mouse plasma with a stock solution of analyte to achieve a nominal concentration from 0.08 µg/mL to 80 µg/mL for both analyte.

A sample volume of 20 µL was transferred into a plastic tube and mixed with 300µL acetonitrile/methanol mixture (3:1, v/v) to precipitate proteins. The tubes was vortexed to mix and centrifuged at 10000 rpm for 10 min. 200 µL of supernatant was diluted with 600 µL of 0.1% formic acid and subjected to LC/MS/MS analysis. Samples above the upper limit of calibration should be diluted 10 times with blank plasma extract and reanalyzed. Bioanalysis of samples from each mouse were measured in duplicate and the results averaged.

In vivo efficacy

Bacterial strains and inoculum. *E. coli* EC 106-09 (EN122) and *K. pneumoniae* SSI #3010 (EN124) are clinical isolates from Denmark. Bacterial suspensions for inoculation of mice were prepared

at room temperature from fresh overnight cultures on 5% blood agar plates produced by SSI diagnostica (Copenhagen Denmark). The inoculum was prepared immediately before inoculation by suspending colonies in sterile 0.9% saline to an optical density of 0.13 at 540 nm, giving a density of 10^8 CFU/ml and further diluted in 0.9% saline to 2 x 10^6 CFU/ml before injecting into the mice. For each experiment, the size of the inoculum was determined by making ten-fold dilutions in 0.9% saline, of which 20 µl was plated on 5% blood agar plates with subsequent counting of colonies after incubation overnight at 35°C in ambient air.

Mice. Outbred female CD-1 mice 26-30 gram (Envigo, Netherlands) were housed at the animal facility at Statens Serum Institut. The temperature was 22°C +/- 2 °C and the humidity was 55 +/- 10%. The mice were housed in individually ventilated type 3 macrolone cages with bedding from Tapvei. The air changes per hour were approximately 8-12 times (70-73 times per hours inside cages), and light/dark period was in 12-hours interval of 6 a.m.- 6 p.m./6 p.m – 6 a.m. The mice had ad libitum access to domestic quality drinking water and food Teklad Global diet 2916C-Envigo) and occasionally peanuts and sunflower seeds (Køge Korn A/S). Further, the animals were offered Enviro-Dri nesting material and cardboard houses (Bio-serv).

Mouse peritonitis model. The mice were rendered neutropenic by injecting 0.5 mL cyclophosphamide solution, 200 mg/kg intraperitoneally at 4 days and 100 mg/kg at 1 day prior to inoculation. 1 h before inoculation, mice were treated orally with 45 ml Nurofen (20 mg ibuprofen/mL corresponding to approximately 30 mg/kg) as a pain relief. Inoculation was performed by intraperitoneal injection of 0.5 ml of the bacterial suspension containing log 6 CFU. The mice were treated subcutaneously with 0.25 – 0.30 mL based on weight, in the neck region with a single dose of compound at 1 hour post inoculation. Mice were monitored for clinical signs of discomfort. 4 h after treatment the mice were anesthetized and blood was collected by heart puncture into EDTA tubes and thereafter mice were euthanized by cervical dislocation and peritoneal washes were performed by injecting 2 ml of sterile saline intraperitoneally, followed by gentle massage of the abdomen and opening the peritoneum to collect fluid. Full blood and peritoneal fluids were serially diluted (ten-fold dilutions) and 20 µl was plated on blood agar plates produced at the SSI diagniostica (Copenhagen, Denmark) with subsequent counting of colonies after incubation overnight at 35°C in ambient. All animal experiments were approved by the National Committee of Animal Ethics – Animal Experiment Inspectorate under the Danish Ministry of Justice.

Statistical analyses. Data was analysed with Dunnett´s multiple comparisons test in GraphPad-Prism 8.3.0. Values of $P< 0.05$ were considered significant.

Chemistry

All chemicals and solvents were purchased from Sigma Aldrich, Fisher Scientific, FluoroChem, Enamine and Ark Pharm chemicals and were used without further purification. Analytical thin-layer chromatography (TLC) was performed using Merck aluminum sheets precoated with silica gel 60 F_{254}. Column chromatography was performed on Merck silica gel 60 (40-63 µm) ^{1}H and ^{13}C NMR spectra were recorded on Varian Mercury Plus instruments; ^{1}H at 399.9 MHz and ^{13}C at 100.6 MHz at 25 ˚C. Chemical shifts (δ) are reported in ppm and coupling constants (*J*) are

reported in hertz (Hz). Exact molecular masses were determined on Micromass Q-Tof2 mass spectrometer equipped with an electrospray ion source. Analytical RP-HPLC-MS was performed on a Gilson RP-HPLC system with a Finnigan AQA quadrupole low-resolution mass spectrometer in positive or negative ESI mode using an Onyx Monolithic C_{18} 3 × 50 mm, 2.6 µm particle size, 100 Å pore size (Phenomenex) with gradients of MeCN in 0.05% aqueous HCOOH as mobile phase at a flow rate of 2 mL/min. Preparative RP-HPLC was performed on a system equipped with a Nucleodur C18 HTec 5 µm column (150 × 21.2 mm) or a Phenomenex C8 5 µm column (150 × 21.2 mm), using a H_2O/MeCN gradient with 0.1% TFA or H_2O/MeCN gradient, flow rate 14 mL/min, using UV detection at 220 nm and 254 nm. High-resolution molecular masses (HRMS) were determined on a Waters acuity UPLC mass spectrometer with an ESI source, 7-T hybrid linear ion trap (LTQ), flow rate 0.25 mL/min (MeCN/H_2O 1:1). Compounds have been characterized by ^{1}H NMR and ^{13}C NMR and ESI (MS). All final compounds were ≥ 95% pure as determined by analytical HPLC and ^{1}H NMR. HPLC purity assessment was done on Waters 2695 equipped with XBridge C18 5 um column (4.6 x 150 mm) using 15 min gradient elution from 5% to 100% MeCN in 0.1% H3PO4 and UV detection at 254 nm (Waters 2487).

Preparation of EBL-3647

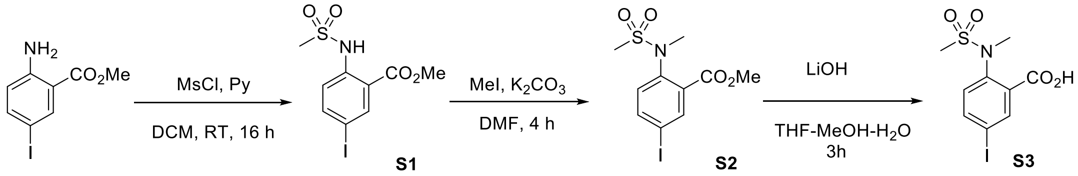

Methyl 5-iodo-2-(methylsulfonamido)benzoate (S1)

Intermediate S1 has been made as previously reported (15). To a solution of methyl 2-amino-5-iodobenzoate (18.7 g, 67.3 mmol) and pyridine (326 ml, 405 mmol) in DCM (95 ml) at ice bath temperature methanesulfonyl chloride (7.84 ml, 101 mmol) was added dropwise over 30 min and the reaction mixture was stirred at room temperature for 16 h. Then HCl (1 M aqueous, 200 ml) was added and the mixture was extracted with DCM (200 ml). Organic layer was extracted with water, dried over Na_2SO_4, and concentrated in vacuo to give compound **S1** (23.9 g, quant.), which was used in the next step without additional purification.

LC/MS (ESI) m/z: 356 $[M+H]^+$.

^{1}H NMR ($CDCl_3$) δ 10.37 (s, 1H), 8.35 (d, *J* = 2.2 Hz, 1H), 7.82 (ddd, *J* = 8.8, 2.2, 0.4 Hz, 1H), 7.51 (d, *J* = 8.8 Hz, 1H), 3.94 (s, 3H), 3.06 (s, 3H).

Methyl 5-iodo-2-(*N*-methylmethylsulfonamido)benzoate (S2)

To a mixture of compound **S1** (18.8 g, 52.9 mmol) and K_2CO_3 (43.9 g, 318 mmol) in DMF (120 ml) MeI (16.5 ml, 265 mmol) was added and the reaction mixture was stirred at room

temperature for 4 h. HCl (1M aqueous, 900 ml) and brine (500 ml) were added and the mixture was extracted with EtOAc (3×250 ml). The EtOAc extracts were combined, extracted with water (400 ml), dried over Na_2SO_4 and evaporated to give compound **20** (19.5 g, quant) which was used in the next step without additional purification.

LC/MS (ESI) m/z: 370 $[M+H]^+$.

^{1}H NMR ($CDCl_3$) δ 8.22 (d, *J* = 2.2 Hz, 1H), 7.86 (dd, *J* = 8.4, 2.2 Hz, 1H), 7.18 (d, *J* = 8.4 Hz, 1H), 3.92 (s, 3H), 3.27 (s, 3H), 2.96 (s, 3H).

5-Iodo-2-(*N*-methylmethylsulfonamido)benzoic acid (S3)

Compound **S2** (12.5 g, 35.1 mmol) and LiOH (4.32 g, 180 mmol) were mixed in THF/water/MeOH, 3:2:1 (300 ml) and the obtained reaction mixture was stirred at room temperature for 3h. The mixture was filtered from undissolved LiOH and the low boiling volatiles were evaporated. Then HCl (0.5 M aqueous, 330 ml) and brine were added to the residue and the mixture was extracted with EtOAc (3×200 ml). The organic extracts acts were combined, extracted with HCl (0.5 M aqueous 100 ml), water (100 ml), brine (100 mL), dried over Na_2SO_4 and evaporated to give acid **S3** (12.5 g, 97% over 3 steps) as a white solid.

LC/MS (ESI) m/z: 356 $[M+H]^+$.

^{1}H NMR ($CDCl_3$) δ 8.02 (d, *J* = 2.2 Hz, 1H), 7.94 (dd, *J* = 8.4, 2.2 Hz, 1H), 7.34 (d, *J* = 8.4 Hz, 1H), 3.19 (s, 3H), 3.00 (s, 3H).

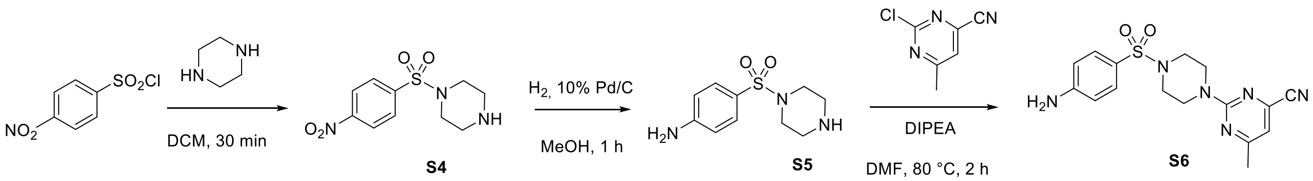

1-((4-Nitrophenyl)sulfonyl)piperazine (S4)

4-Nitrobenzenesulfonyl chloride (**12**) (10.00 g, 45.1 mmol) was added in one portion to a solution of piperazine (38.9 g, 451 mmol) in DCM (450 ml) at 0 °C and the reaction mixture was stirred at 0 °C for 30 min. DCM (200 ml) was added and the mixture was extracted, with saturated $NaHCO_3$ (200 mL) solution, water (2×200 ml), brine (2x200 ml), dried (Na_2SO_4), and concentrated in vacuo to give compound **13** (10.1 g, 82%) as a yellow solid, which was used in the next step without additional purification.

LC/MS (ESI) m/z: 272 $[M+H]^+$.

^{1}H NMR (DMSO-d_6) δ 8.48 – 8.43 (m, 2H), 8.02 – 7.96 (m, 2H), 3.92-2.83 (m, 4H), 3.78-2.70 (m, 4H).

4-(Piperazin-1-ylsulfonyl)aniline (S5)

To a solution of compound **S4** (9.90 g, 36.5 mmol) in MeOH (400 ml) 10% Pd/C (2.00 g, 1.9 mmol) was added and the resulting mixture was stirred under H_2 atmosphere at room temperature for 2 h. The catalyst was filtered off through a pad of Celite, the pad was rinsed with MeOH (100 mL). The filtrate was concentrated in vacuo to give compound **14** (8.35 g, 94%) as a white solid, which was used in the next step without additional purification.

LC/MS (ESI) m/z: 242 $[M+H]^+$.

^1H NMR ($CDCl_3$) δ 7.58 – 7.46 (m, 2H), 6.74 – 6.63 (m, 2H), 4.12 (s, 2H), 2.98-2.88 (m, 8H), 1.47 (br s, 1H).

2-(4-((4-Aminophenyl)sulfonyl)piperazin-1-yl)-6-methylpyrimidine-4-carbonitrile (S6)

2-Chloro-6-methyl-pyrimidine-4-carbonitrile (6.00 g, 3.91 mmol) was added to a solution of piperazine derivative **S5** (9.90 g, 41.0 mmol) and DIPEA (8.17 ml, 4.69 mmol) in DMF (90 mL). The reaction mixture was stirred at 80°C for 4 h and then was poured into water (300 ml). After stirring at room temperature for 30 min precipitates were filtered, washed with water(2×100 ml) and Et_2O (50 mL) , dried in vacuo to give compound **S6** (13.1 g, 93%) as slightly orange solid.

LC/MS (ESI) m/z: 359 $[M+H]^+$.

^1H NMR ($CDCl_3$) δ 7.56 – 7.50 (m, 2H), 6.71 – 6.65 (m, 2H), 6.64 (s, 1H), 4.12 (s, 2H), 4.00 – 3.87 (m, 4H), 3.07 – 2.96 (m, 4H), 2.35 (s, 3H).

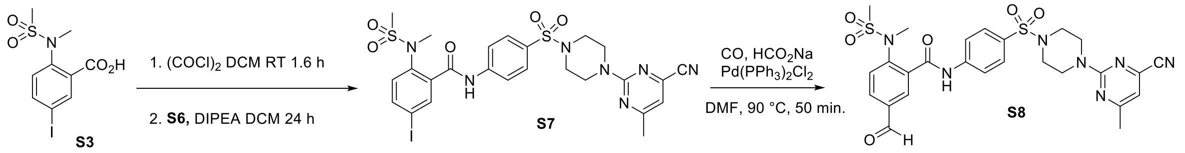

***N*-(4-((4-(4-Cyano-6-methylpyrimidin-2-yl)piperazin-1-yl)sulfonyl)phenyl)-5-iodo-2-(*N*-methylmethylsulfonamido)benzamide (S7)**

To a suspension of carboxylic acid derivative **S3** (1.50 g, 4.2 mmol) in DCM (30 ml), oxalyl chloride (0.89 ml, 10.5 mmol) were added followed by addition of a drop of DMF. The reaction mixture was stirred at room temperature for 1.6 h. Volatiles were removed under reduced pressure and the residue was redissolved in DCM (20 mL). This solution was added dropwise to a cold (ice bath) solution of aniline **S6** (1.26 g, 3.5 mmol) and DIPEA (1.52 ml, 8.8 mmol) in DCM (30 mL). The reaction mixture was stirred at room temperature for 24 h. DCM (100 ml) was added and the mixture was extracted with water (2×20 mlNaHCO$_3$ (5%, aqueous, 2×20 ml) solution, brine (2×50 ml), dried (Na_2SO_4), and evaporated to dryness. The residue was purified

by column chromatography on silica gel (eluent hexanes/EtOAc from 1:1 to 1:1.5) to afford compound **S7** (2.17 g, 89%) as a white solid.

LC/MS (ESI) m/z: 696 $[M+H]^+$.

^{1}H NMR ($CDCl_3$) δ 9.41 (s, 1H), 8.14 (d, *J* = 2.2 Hz, 1H), 7.88 (dd, *J* = 8.4, 2.2 Hz, 1H), 7.86 – 7.81 (m, 2H), 7.76 – 7.71 (m, 2H), 7.08 (d, *J* = 8.4 Hz, 1H), 6.64 (s, 1H), 4.00-3.93 (m, 4H), 3.25 (s, 3H), 3.10 (s, 3H), 3.08-3.03 (m, 4H), 2.35 (s, 3H).

***N*-(4-((4-(4-Cyano-6-methylpyrimidin-2-yl)piperazin-1-yl)sulfonyl)phenyl)-5-formyl-2-(*N*-methylmethylsulfonamido)benzamide (S8)**

Iodide **S7** (2.00 g 2.88 mmol) and $Pd(PPh_3)_2Cl_2$ (210 mg, 4.31 mmol, 10 mol %) were dissolved in DMF (7 ml). Carbon monoxide was bubbled through the mixture for 15 min. Then the reaction mixture was stirred at 90°C for 50 min. After cooling to room temperature EtOAc (100 mL) was added, and the mixture was extracted with water (3×50 ml), sat. $NaHCO_3$ solution (2×50 ml), brine (50 ml) and dried over Na_2SO_4. Obtained EtOAc solution was filtered through a short column with silica gel and evaporated. The residue was purified by column chromatography on silica gel (eluent DCM/acetone, gradient from 2 to 18%) and product containing fraction were evaporated to dryness. The residue was suspended in DCM (20 ml), the suspension kept in the refrigerator overnight, and filtered. The solid was dried in vacuo to give compound **S8** (840 mg, 49%) as a white solid.

LC/MS (ESI) m/z: 598 $[M+H]^+$.

^{1}H NMR (DMSO) δ 10.85 (s, 1H), 10.09 (s, 1H), 8.13 (d, *J* = 2.0 Hz, 1H), 8.11 (dd, *J* = 8.1, 2.0 Hz, 1H), 7.99 – 7.90 (m, 2H), 7.83 (d, *J* = 8.1 Hz, 1H), 7.79 – 7.70 (m, 2H), 7.10 (s, 1H), 3.90-3.78 (m, 4H), 3.28 (s, 3H), 3.05 (s, 3H), 3.02-2.92 (m, 4H), 2.33 (s, 3H).

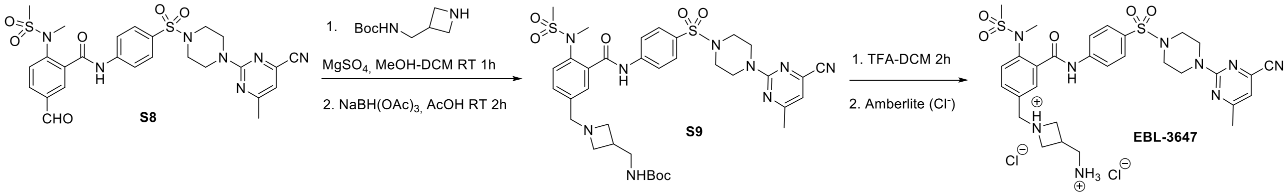

***tert*-Butyl ((1-(3-((4-((4-(4-cyano-6-methylpyrimidin-2-yl)piperazin-1-yl)sulfonyl)phenyl)carbamoyl) -4-(*N*-methylmethylsulfonamido)benzyl)azetidin-3-yl)methyl)carbamate (S9)**

A mixture of aldehyde **S8** (902 mg, 1.51 mmol) and *tert*-butyl *N*-(azetidin-3-ylmethyl)carbamate (560 mg, 300 mmol) in in DCM (10 mL) was stirred at room temperature for 1h. $NaBH(OAc)_3$ (384 mg, 1.81 mmol) was added and the mixture was stirred for 2h.

DCM (200 ml) was added, and the mixture was extracted with saturated $NaHCO_3$ solution (100 mL), brine (100 mL), dried over Na_2SO_4, and evaporated. The residue was purified by column chromatography (eluent DCM/acetone=10:1) to give compound **S9** as a transparent amorphous solid (1.02 g, 88%).

LC/MS (ESI) m/z: 769 $[M+H]^+$.

^1H NMR ($CDCl_3$) δ 9.58 (s, 1H), 7.94 – 7.85 (m, 2H), 7.80 – 7.70 (m, 3H), 7.50 (dd, *J* = 8.2, 2.2 Hz, 1H), 7.33 (d, *J* = 8.2 Hz, 1H), 6.64 (s, 1H), 4.81 (br s, 1H), 4.02-3.93 (m, 4H), 3.75 (s, 2H), 3.55-3.45 (m, 2H), 3.36-3.27 (m, 2H), 3.26 (s, 3H), 3.20-3.11 (m, 2H), 3.10 (s, 3H), 3.08-3.03 (m, 4H), 2.79-2.63 (m, 1H), 2.35 (s, 3H), 1.43 (s, 9H).

3-(Ammoniomethyl)-1-(3-((4-((4-(4-cyano-6-methylpyrimidin-2-yl)piperazin-1-yl)sulfonyl)phenyl) carbamoyl)-4-(*N*-methylmethylsulfonamido)benzyl)-2,3-dihydroazet-1-ium dichloride (EBL-3647)

To a solution of compound **S9** (56.0 mg, 0.0729 mmol) in dry DCM (2 ml) under argon at ice bath temperature, TFA (0.5 ml) was added dropwise and the reaction mixture was stirred for 2 h during which it was allowed gradually warm up to room temperature. The reaction mixture was diluted with DCM (5 ml), the volatiles evaporated, and the residue was co-evaporated with DCM three times. The residue was dissolved in water (0.5 ml) and passed through an ion-exchange resin (Amberlite IRA-410 (Cl), 5 ml) column (eluent water). The eluate containing product was freeze dried to give compound **EBL-3647** as white fluffy solid (54 mg, 97%).

LC/MS (ESI) m/z: 669 $[M+H]^+$.

^1H NMR (DMSO-d_6) δ 11.61 – 11.29 (m, 1H), 10.78 – 10.74 (m, 1H), 8.08 (s, 3H), 7.96 (d, *J* = 8.9 Hz, 2H), 7.82 – 7.79 (m, 1H), 7.79 – 7.76 (m, 1H), 7.74 (d, *J* = 8.9 Hz, 2H), 7.71 – 7.66 (m, 1H), 7.10 (s, 1H), 4.46 (d, *J* = 6.0 Hz, 2H), 4.23 – 4.14 (m, 1H), 4.04 – 3.97 (m, 2H), 3.96 – 3.90 (m, 1H), 3.89 – 3.78 (m, 4H) 3.30 – 3.20 (m, 5H), 3.13 – 3.06 (m, 1H), 3.00 (s, 3H) 2.99-2.97 (m, 4H), 2.33 (s, 3H).

^{13}C NMR (DMSO-d_6) 171.2, 166.1, 160.8, 143.9, 140.6, 140.1, 137.2, 133.2, 131.3, 131.2, 130.9, 130.9, 129.5, 129.3, 119.9, 116.9, 113.4, 56.7, 56.3, 56.1, 55.6, 46.0, 43.0, 40.8, 38.9, 37.2, 37.2, 27.7, 27.4, 24.5.

HRMS-ESI(+) (m/z): [M+H]+ calcd for $C_{30}H_{38}N_9O_5S_2$, 668.2437, found: *m/z* 668.2451

Preparation of EBL-3599

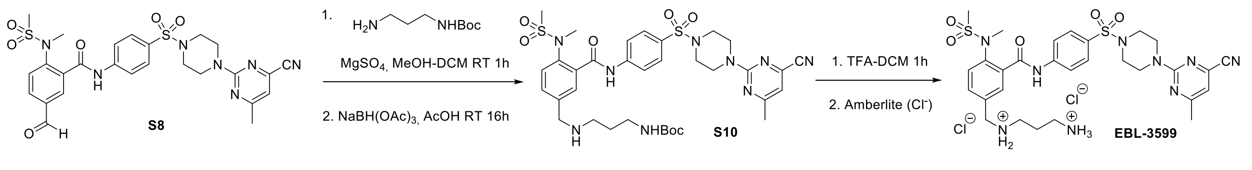

***tert*-Butyl (3-((3-((4-((4-(4-cyano-6-methylpyrimidin-2-yl)piperazin-1-yl)sulfonyl)phenyl)carbamoyl) -4-(*N*-methylmethylsulfonamido)benzyl)amino)propyl)carbamate (S10)**

To a suspension of aldehyde **S8** (820 mg, 1.37 mmol) in DCM (10 ml), tert-butyl (3-aminopropyl)carbamate (1.20 g, 6.86 mmol) and acetic acid (0.16 ml, 2.75 mmol) were added, and the mixture was stirred at room temperature for 1h. $NaBH(OAc)_3$ (349 mg, 1.65 mmol) was added and the mixture was stirred for 16h. Additional amount of $NaBH(OAc)_3$ (450 mg, 2.12 mmol) was added portionwise and the mixture was stirred for 16h. DCM (200 ml) was added, and the mixture was extracted with saturated $NaHCO_3$ solution (100 mL), brine (100 mL), dried over Na_2SO_4, and evaporated. The residue was purified by column chromatography (eluent EtOAc/MeOH, gradient from 0 to 100%) to give compound **S10** as a white foam (655 mg, 63%).

LC/MS (ESI) m/z: 757 $[M+H]^+$.

^1H NMR (400 MHz, $CDCl_3$) δ 9.59 (s, 1H), 7.88 (d, *J* = 8.4 Hz, 2H), 7.78 (s, 1H), 7.76 – 7.71 (m, 2H), 7.56 (d, *J* = 8.3 Hz, 1H), 7.31 (d, *J* = 8.3 Hz, 1H), 6.64 (s, 1H), 4.95 (br s, 1H), 4.01-3.93 (m, 4H), 3.84 (s, 2H), 3.26 (s, 3H), 3.27-3.19 (m, 2H), 3.10 (s, 3H), 3.09-3.01 (m, 4H), 2.71 (t, *J* = 6.4 Hz, 2H), 2.35 (s, 3H), 1.86 (br s, 1H), 1.68 (p, *J* = 6.7 Hz, 2H), 1.42 (s, 9H).

N^1-(3-((4-((4-(4-cyano-6-methylpyrimidin-2-yl)piperazin-1-yl)sulfonyl)phenyl)carbamoyl)-4-(*N*-methylmethylsulfonamido)benzyl)propane-1,3-diaminium dichloride (EBL-3599)

To a solution of compound **S10** (600 mg, 0.794 mmol) in dry DCM (6 ml) under argon at ice bath temperature, TFA (2 ml) was added dropwise and the reaction mixture was stirred for 1 h during which it was allowed gradually warm up to room temperature. The reaction mixture was diluted with DCM (5 ml), the volatiles evaporated, and the residue was co-evaporated with DCM three times. Then the residue was dissolved in water (2 ml) and passed through an ion-exchange resin (Amberlite IRA-410 (Cl), 30 ml) column (eluent water). The eluate containing product was freeze dried to give yellow fluffy solid (560 mg). The solid was dissolved in water (2ml), purified on reverse phase chromatography (eluent 0.1% HCOOH in water/CH_3CN, 0 - 50%). The eluate containing product was freeze dried to give compound **EBL-3559** as white fluffy solid (382 mg, 66%).

LC/MS (ESI) m/z: 656 $[M+H]^+$.

^1H NMR (DMSO-d_6) δ 7.95 (d, *J* = 8.7 Hz, 2H), 7.90 (d, *J* = 2.3 Hz, 1H), 7.82 – 7.75 (m, 3H), 7.70 (d, *J* = 8.2 Hz, 1H), 6.86 (s, 1H), 4.35 (s, 2H), 3.93 (t, *J* = 5.1 Hz, 4H), 3.34 (s, 3H), 3.22 (t, 2H), 3.11 – 3.03 (m, 6H), 3.02 (s, 3H), 2.37 (s, 3H), 2.20 – 2.09 (m, 2H).

^{13}C NMR (DMSO-d_6) 172.26, 167.77, 162.37, 144.29, 142.33, 141.65, 138.44, 134.48, 132.64, 132.18, 130.31, 130.11, 121.32, 117.31, 113.87, 51.55, 47.11, 45.77, 44.25, 39.24, 37.89, 36.96, 25.40, 24.30.

HRMS-ESI(+) (m/z): [M+H]+ calcd for $C_{29}H_{38}N_9O_5S_2$, 656.2437, found: *m/z* 656.2451

Preparation of EBL-3025

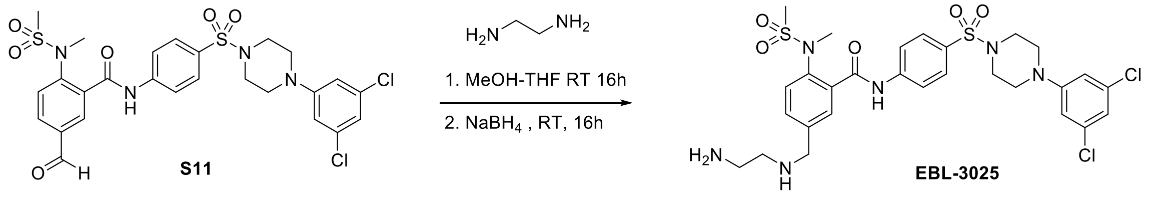

5-(((2-Aminoethyl)amino)methyl)-*N*-(4-((4-(3,5-dichlorophenyl)piperazin-1-yl)sulfonyl)phenyl)-2-(*N*-methylmethylsulfonamido)benzamide (EBL-3025)

30mg of *N*-(4-((4-(3,5-dichlorophenyl)piperazin-1-yl)sulfonyl)phenyl)-5-formyl-2-(*N*-methylmethyl- sulfonamido)benzamide **S11** (prepared in a same way as described for **S8**) was dissolved in methanol/THF (total volume about 2 ml) and ethane-1,2-diamine (23 mg) was added followed by stirring overnight at r.t. Sodium borohydride (7 mg, 3 eq) was added. Reaction mixture was stirred for about 24h at r.t., concentrated by rotary evaporation, mixed with water and extracted with ethyl acetate. Dried organic extracts were concentrated and purified by prep HPLC using 15 min gradient 30 -80 % acetonitrile/water with 0.05% of formic acid, C18 column, flowrate 10 ml/min. Yield: 15 mg (white fluffy powder after freeze drying).

LC/MS (ESI) m/z: $[M+H]^+$ 669.

^{1}H NMR (CD_3CN) δ 10.63 (s, 1H), 8.22 (s, 2H), 7.98 (m, 2H), 7.74 (m, 3H), 7.45 (m, 2H), 6.82 (m, 3H), 3.26 (d, 8H), 3.03 (m, 4H), 2.94 (m, 5H), 2.79 (m, 2H);

^{13}C NMR (CD_3CN) δ 153.37, 144.77, 141.85, 138.64, 137.77, 136.13, 132.23, 130.36, 130.09, 130.00, 129.78, 129.33, 120.60, 119.45, 115.06, 52.72, 48.51, 47.17, 46.66, 40.29, 39.63, 37.42.

HRMS-ESI(+) (m/z): [M+H]+ calcd for $C_{28}H_{35}Cl_2N_6O_5S_2$, 669.1487, found: *m/z 669.1475*

Preparation of EBL-3339

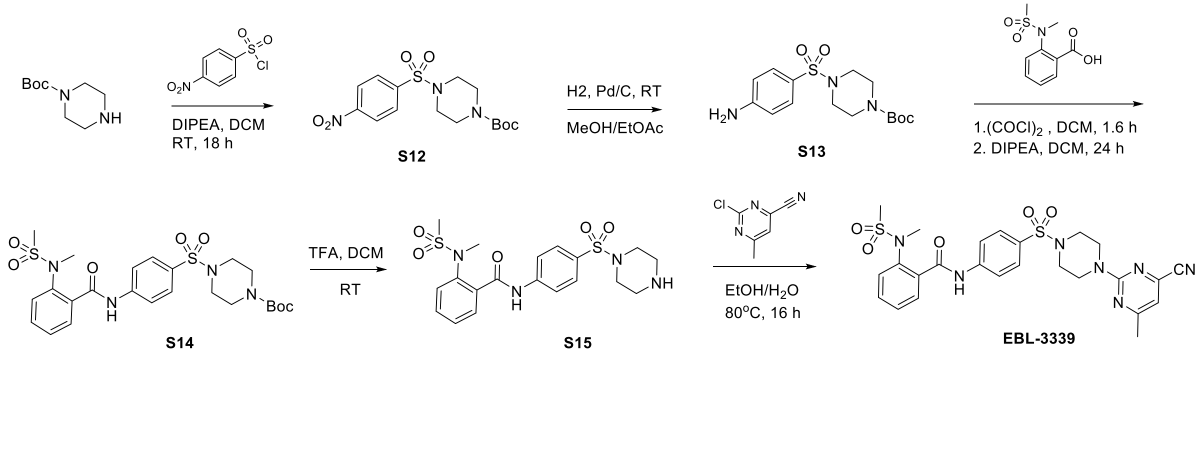

***tert*-butyl 4-(4-nitrophenyl) sulfonyl) piperazine-1-carboxylate (S12)**

A mixture of p-nitrobenzenesulfonyl chloride (4.28 g, 1.2 eq.), tert-butyl piperazine-1-carboxylate (3.0 g, 1.0 eq.) and 4.2 ml (1.5 eq.) of diisopropylethylamine in 30 ml of tetrahydrofuran was stirred at room temperature for 18 hours. The solvent was then removed in vacuo and the residue was extracted with methylene chloride/aqueous sodium bicarbonate and brine. The organic layer was filtered through anhydrous sodium sulfate bed and taken to dryness. Crude product was purified by column chromatography on silica gel (10% EtOAc in hexanes) to yield the title product as solid (quantitative yield).

LC/MS (ESI) m/z: 372 $[M+H]^+$.

^{1}H NMR ($CDCl_3$) δ 8.33 (m, 2H), 7.88 (m, 2H), 3.46 (m, 4H), 2.97 (m, 4H), 1.34 (s, 9H).

***tert*-butyl 4-(4-aminophenyl)sulfonylpiperazine-1-carboxylate (S13)**

3.0 g of tert-butyl 4-(4-nitrophenyl) sulfonyl) piperazine-1-carboxylate **(S12)** were dissolved in 100 mL of 1:1 mixture of methanol and EtOAc and 0.3 g. of 10% palladium on charcoal was added to it under nitrogen. The flask was then evacuated and filled with hydrogen, this process repeated three times and the hydrogenation was allowed to proceed until the nitro compound consumed completely. The time required for hydrogenation is approximately 12 hours. After venting the hydrogen from the flask safely, the solution filtered through Celite bed and the catalyst washed carefully with methanol. The filtrate was transferred to a flask which was placed on a rotary evaporator and removed the solvent at reduced pressure, affording (99% yield) of colourless product.

LC/MS (ESI) m/z: 342 $[M+H]^+$.

^{1}H NMR ($CDCl_3$) δ 7.50 (d, 2H), 6.72 (d, 2H), 4.17 brs, 2H), 3.48 (m, 4H), 2.92 (m, 4H), 1.40 (s, 9H).

***tert*-butyl 4-((4-(2-(*N*-methylmethylsulfonamido)benzamido)phenyl)sulfonyl)piperazine-1-carboxylate (S14)**

Oven dried round bottom flask, under nitrogen was charged with 1.0 eq., of 2-[methyl (methylsulfonyl)amino] benzoic acid, 10 ml of CH_2Cl_2 and 1.0 ml of thionyl chloride. The mixture was refluxed for 3.0 hours. Then the solvent and remaining thionyl chloride were distilled off. Fresh DCM was added and distilled off again. The resulted acid chloride was taken into 10 ml of dry DCM and TEA (3.0 eq) was added to it at 0-5 ^oC under nitrogen atmosphere. To this mixture, a solution of tert-butyl 4-(4-aminophenyl) sulfonylpiperazine-1-carboxylate **S13** (0.9 eq) in dry DCM was added dropwise and allowed to stir at RT overnight. Then reaction was diluted with DCM and the organic layer was washed with water and finally with brine. Dried over $MgSO_4$ and concentrated by rotary evaporation to obtain the crude product which was purified by column chromatography on silica (20% EtOAc in hexanes; 82 % yield).

LC/MS (ESI) m/z: 453 $[M+H]^+$.

^{1}H NMR ($CDCl_3$) δ 9.55 (s, 1H), 7.88 (m, 2H), 7.83 (m, 1H), 7.72 (m, 2H), 7.54 (m, 2H), 7.36 (m, 1H), 3.49 (m, 4H), 3.28 (s, 3H), 3.12 (s, 3H), 2.97 (m, 4H), 1.40 (s, 9H).

2-(*N*-methylmethylsulfonamido)-*N*-(4-(piperazin-1-ylsulfonyl)phenyl)benzamide (S15)

To a solution of tert-butyl 4-[4-[[2-[methyl(methylsulfonyl)amino]benzoyl]amino]phenyl] sulfonylpiperazine-1-carboxylate **(S14)** in dichloromethane was added trifluoroacetic acid (5.0 eq) at room temperature dropwise and the resultant reaction mixture was stirred at RT for 3.0 hrs. Then removed solvent under reduced pressure and the residue was taken into DCM and washed with aqueous $NaHCO_3$ solution. Organic layer was dried over MgSO4 and concentrated to give the title product which was pure enough and used in the next step without any purification.

LC/MS (ESI) m/z: 453 $[M+H]^+$.

^{1}H NMR ($CDCl_3$) δ 9.51 (s, 1H), 7.85 (m, 3H), 7.71 (m, 2H), 7.55 (m, 2H), 7.37 (m, 1H), 3.28 (s, 3H), 3.12 (s, 3H), 3.02 (dd, 4H), 2.95 (dd, 4H), 2.85 (brs, 1H).

***N*-(4-((4-(4-cyano-6-methylpyrimidin-2-yl)piperazin-1-yl)sulfonyl)phenyl)-2-(*N*-methylmethylsulfonamido)benzamide (EBL-3339)**

2-(*N*-methylmethylsulfonamido)-*N*-(4-(piperazin-1-ylsulfonyl)phenyl)benzamide (50 mg) and 2-chloro-6-methyl-pyrimidine-4-carbonitrile (51 mg, 3 eq) were dissolved in ethanol (1.5 ml) and water (0.2 ml) mixture and heated at stirring in a closed vial at 80 °C for about 16h. Reaction mixture was mixed with water and extracted into ethylacetate (4x20 ml), washed with brine, dried over magnesium sulfate. Organic extract was concentrated by rotary evaporation and purified by column chromatography on silica gel. Fractions containing desired material were concentrated and freeze dried to give 18 mg of fluffy white powder.

LC/MS (ESI) m/z: 570 $[M+H]^+$.

^{1}H NMR ($CDCl_3$) δ 9.51 (s, 1H), 7.93 – 7.79 (m, 3H), 7.79 – 7.67 (m, 2H), 7.63 – 7.46 (m, 2H), 7.36 (dd, 1H), 6.64 (s, 1H), 4.05 – 3.85 (m, 4H), 3.27 (s, 3H), 3.12 (s, 3H), 3.05 (m, 4H), 2.35 (s, 3H);

^{13}C NMR ($CDCl_3$) δ 170.37, 165.46, 160.88, 142.51, 141.35, 137.82, 137.26, 132.27, 131.47, 130.31, 129.89, 129.26, 127.16, 119.95, 116.39, 112.74, 46.00, 43.10, 40.10, 38.96, 24.50.

HRMS-ESI(+) (m/z): [M+H]+ calcd for $C_{25}H_{28}N_7O_5S_2$, 570.1593, found: *m/z 570.1604*

Preparation of JEDI-1444

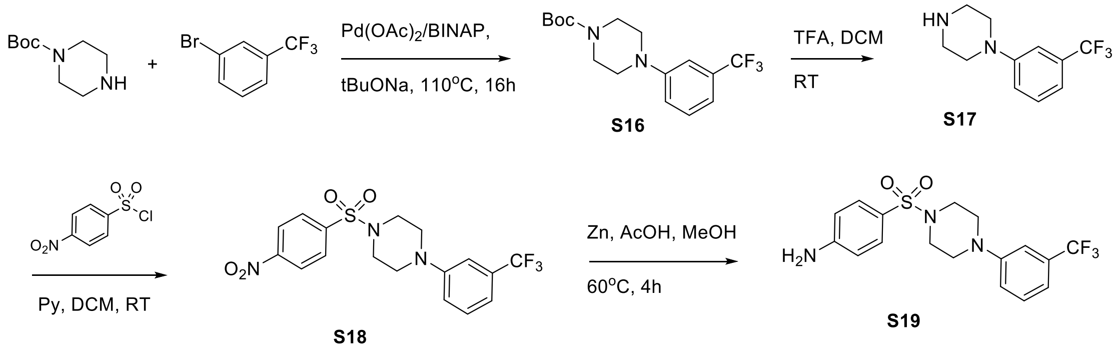

tert-butyl 4-[3-(trifluoromethyl) phenyl] piperazine-1-carboxylate (S16)

To a mixture of tert-butyl piperazine-1-carboxylate (1.0 g, 5.37 mmol), 1-bromo-3-(trifluoromethyl)- benzene (1.0 mL, 7.34 mmol), BINAP (0.135 g, 0.22 mmol), and NaOt-Bu (0.670 g, 6.57 mmol) in toluene (20 mL) was added Pd $(OAc)_2$ (0.024 g, 0.11 mmol). The mixture was heated at 110 °C for 16.0hrs, then allowed to cool to room temperature. The mixture was filtered over Celite, and the filter cake was washed with EtOAc. The filtrate was concentrated under reduced pressure, and the resulting residue was purified by flash column chromatography (5% EtOAc in hexanes) to give tert-butyl 4-(3- (trifluoromethyl)-phenyl) piperazine-1-carboxylate as a pale-yellow solid (80% in yield).

LC/MS (ESI) m/z: 331 $[M+H]^+$.

^{1}H NMR ($CDCl_3$) δ 7.29 (m, 1H), 7.03 (m, 3H), 3.53 (m, 4H), 3.11 (m, 4H), 1.41 (s, 9H).

1-[3-(Trifluoromethyl) phenyl]piperazine (S17)

To a solution of *tert*-butyl 4-[3-(trifluoromethyl) phenyl] piperazine-1-carboxylate **(S16)** in dichloromethane was added trifluoroacetic acid (5.0 eq) at room temperature dropwise and the resultant reaction mixture was stirred at RT for 2.0 hrs. Then removed solvent under reduced pressure and the residue was taken into DCM and washed with aqueous NaHCO3 solution. Organic layer was dried over MgSO4 and concentrated to give the title Product which is pure enough and used in the next step without any purification.

LC/MS (ESI) m/z: 231 $[M+H]^+$.

^{1}H NMR ($CDCl_3$) δ 7.27 (t, 1H), 7.00 (m, 3H), 3.11 (m, 4H), 2.97 (m, 4H), 2.03 (brs, 1H).

1-(4-Nitrophenyl)sulfonyl-4-[3-(trifluoromethyl)phenyl]piperazine (S18)

A mixture of 1.42 g (6.41 mmol) of p-nitrobenzenesulfonylchloride, 1.23 g (5.34 mmol) of 1-[3-(trifluoromethyl)phenyl]piperazine **(S17)**, 1.4 ml (8.00 mmol) of diisopropylethylamine and 30 ml of tetrahydrofuran was stirred at room temperature for 18 hours. The solvent is then

removed in vacuo and the residue is extracted with methylene chloride/aqueous sodium bicarbonate and brine. The organic layer was filtered through sodium sulfate and taken to dryness. Crude Product was purified by eluting with dichloromethane and methanol (5 ml of methanol in 250 ml of DCM) to yield the title product (90% yield and is a solid).

^{1}H NMR ($CDCl_3$) δ 8.41 (m, 2H), 7.99 (m, 2H), 7.35 (m, 1H), 7.14 (m, 1H), 7.04 (m, 2H), 3.31 (m, 4H), 3.26 (m, 4H); LC/MS (ESI) m/z: 416 $[M+H]^+$.

4-[4-[3-(Trifluoromethyl)phenyl]piperazin-1-yl]sulfonylaniline (S19)

A suspension of nitro compound **(S18)** and Zn (4.0 eq) dust in methanol (30 ml) was stirred with acetic acid (5.0 ml) at 60°C. After 4.0 hours, LCMS showed complete conversion. After completion of the reaction (monitored by TLC and LCMS), the mixture was filtered off. The organic layer was evaporated and the residue was dissolved in EtOAc and washed with water, saturated NaCl. The organic layer upon evaporation gave the desired amine and used in the next step without any purification (97% yield).

LC/MS (ESI) m/z: 386 $[M+H]^+$.

^{1}H NMR ($CDCl_3$) δ 7.56 (m, 2H), 7.34 (t, 1H), 7.07 (m, 3H), 6.71 (m, 2H), 4.12 (brs, 2H), 3.28 (m, 4H), 3.15 (m, 4H).

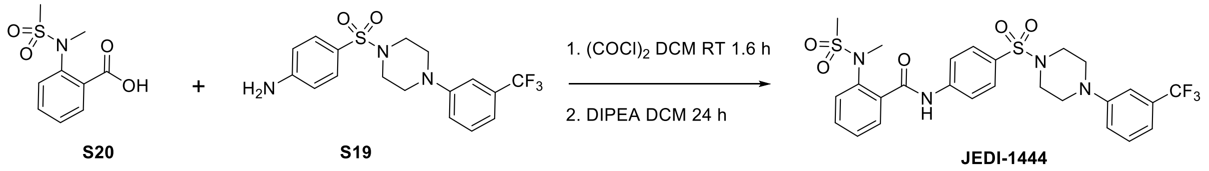

2-[Methyl(methylsulfonyl)amino]benzoic acid (S20)

Compound was prepared from methyl 2-aminobenzoate in a same way as described for the intermediate S3.

LC/MS (ESI) m/z: 228 $[M-H]^-$.

^{1}H NMR ($CDCl_3$) δ 7.98 (m, 1H), 7.55 (m, 1H), 7.41 (m, 2H), 3.28 (s, 3H), 2.92 (s, 3H).

2-(*N*-methylmethylsulfonamido)-*N*-(4-((4-(3-(trifluoromethyl)phenyl)piperazin-1-yl)sulfonyl) phenyl)benzamide (JEDI-1444)

Compound was prepared from 2-[methyl (methylsulfonyl)amino] benzoic acid **S20** (100 mg) and 4-[4-[3-(trifluoromethyl) phenyl] piperazin-1-yl]sulfonylaniline **S19** (151 mg) in a same way as described for the compound **S7**. Recrystallized from EtOAc and hexanes after column chromatography on silica to give desired product (150 mg, 58%) as a white solid.

LC/MS (ESI) m/z: 597 $[M+H]^+$.

^{1}H NMR ($CDCl_3$) δ 9.48 (s, 1H), 7.84 (m, 2H), 7.77 (dd, 1H), 7.70 (m, 2H), 7.48 (m, 2H), 7.28 (m, 2H), 7.01 (m, 3H), 3.22 (d, 7H), 3.12 (dd, 4H), 3.06 (s, 3H);

^{13}C NMR ($CDCl_3$) δ 165.47, 150.82, 142.60, 137.86, 137.24, 132.27, 132.16, 131.85, 131.53, 131.44, 131.21, 130.25, 129.87, 129.30, 127.24, 125.58, 122.87, 119.89, 119.78, 117.20 (m), 113.25 (m), 48.86, 46.00, 40.09, 37.03.

HRMS-ESI(+) (m/z): [M+H]+ calcd for $C_{26}H_{28}N_4O_5S_2F_3$, 597.1453, found: *m/z 597.1448*

Supplementary biochemistry and structural biology results

Goodness of fit to electron density

The fit of the product or inhibitor to the electron density in sigmaA-weighted maps (16) is excellent in each complex. The Maximum Enveloping Electron Density (MEED) score provides a more quantitative measure of the goodness of fit. This indicator allows us to define which bonds are not perfectly enveloped at a particular density value, and the highest density at which each bond ceases to be enveloped. The bonds with a MEED score below the root-mean-square (RMS) of each map are indicated by magenta coloring in *SI Appendix*, Figure S3. In the EcLpxH/lipid X complex (PDB code 8QJZ), one of the aliphatic chains is partially solvent exposed; the resulting disorder means that seven bonds are not enveloped at the RMS of the map. In the EcLpxH/JEDI-1444 complex (PDB code 8QK9), one fluorine of the trifluoromethyl group on ring 1 is outside the RMS boundary, while the other two are on the inside edge. In the KpLpxH/JEDI-852 complex (PDB code 8QKA), the inhibitor is completely enveloped at the RMS of the map, while in KpLpxH/JEDI-3339 (PDB code 8QK2), the nitrogen of the cyano group on ring 1 is near but outside the RMS boundary. In the KpLpxH/JEDI-3647 complex (PDB code 8QK5), the final two bonds of the C5-extension are not enveloped; the relevant atoms are solvent-exposed and make no specific interactions with the enzyme that would order them (*SI Appendix*, Figure S3E).

The overall fit of each enzyme model to its respective electron density is also excellent. There are no significant breaks at the RMS of the map, except for one segment in $\alpha 6$ that is disordered in all enzyme-inhibitor complexes, but is well defined in the enzyme-product complex, as described below. A MEED analysis of the main-chain atoms for each residue in the range 2-240 (defined as C-, N, CA, C, O, N+, where the minus and plus signs indicate atoms from preceding and following residues) shows outliers are limited to the residues immediately before and after the region of disorder in $\alpha 6$ of the inhibitor complexes, except for the carbonyl oxygen of residue 220 in the KpLpxH/JEDI-3647, which is just outside the RMS electron density boundary. His-tag residues have been modelled at the C-terminus where there is appropriate density, but show carbonyl oxygens at the edge of the RMS boundary for KpLpxH complexes with JEDI-852 (His241), JEDI-3339 (His241), and JEDI-3647 (His242, His243).

Product and inhibitor binding sites

The fold observed in the EcLpxH and KpLpxH structures was first described for *Haemophilus influenzae* LpxH (PDB code 5K8K)(17). For example, our EcLpxH product complex can be aligned with the lipid X complexes of *H. influenzae* LpxH and the *Pseudomonas aeruginosa* enzyme (PaLpxH, PDB code 5B49)(18), with pairwise RMSD values of ~1.0 Å for 234/238 $C\alpha$ atoms, respectively, using a 3.8-Å cut-off. A core domain consisting of two tightly-packed central β-sheets decorated on both sides with helices frames an active site containing two metal ions, with coordinating side chains drawn from both sheets (*SI Appendix*, Figure S1A). A large insertion between β6 and $\alpha 8$ in one sheet (residues ~121-173, which includes three α-helices, $\alpha 4$-$\alpha 6$ in the nomenclature of Okada et al. (18) that we adopt throughout the discussion) provides a lid over one edge of the core domain, creating a binding site for a large, complex substrate containing both polar and lipophilic segments. Portions of this lid become disordered in structures of the H10N mutant of PaLpxH(18).

Enzyme-product interactions in EcLpxH are mostly limited to side chains from helices in the lid (α4-6), parts of the loops connecting β2 and α2 (residues 45-47), and β3 and α3 (residues 79-83), from the larger sheet (*SI Appendix*, Figure S1A). Thirty-one residues show a change in solvent-accessible surface area (ASA) (19) on the removal of the product from the model of the complex, which results in a loss of ASA of 619 $Å^2$ for the enzyme alone. For simplicity, we limit ourselves to showing the 19 residues that have direct polar interactions with the head group and those that define the apolar binding surface for the acyl chains (together producing an ASA change of 489 $Å^2$). The glucosamine-1-phosphate head group is locked into position by a number of interactions with enzyme side chains (*SI Appendix*, Figure S1B); notably, the guanidino groups of Arg80 and Arg157 form salt links with the phosphate group of the product (five NH - O hydrogen bonds), and a band of side chains from the long α6 helix (Ser160, Asn164 and Lys167) contribute another seven hydrogen bonds. Two residues that are pre-aligned via coordination to one of the manganese ions, Asn79 and His195, contribute a pair and a single hydrogen bond, respectively. The hydroxyl group on the C2-acyl chain forms an intramolecular hydrogen bond with a phosphate oxygen, and only two polar atoms of the 20 in the product lack hydrogen-bonding partners (both in the C3-acyl chain). Three water molecules complete the set of polar interactions with the head group. The two acyl chains of the product show different degrees of exposure to solvent (*SI Appendix*, Figure S1A, S2D). The C2-acyl chain reaches the surface by protruding in a gap between α4 and α6. The aliphatic tail of this chain has a significant contact surface (ASA of 165 $Å^2$) while packing against mostly hydrophobic side chains from α4 (Phe128) and α6 (Ile152, Met156 and Asn159), which results in relatively poorly-defined electron density (*SI Appendix*, Figure S3A). In contrast, the C3-acyl chain is almost completely buried in the enzyme (ASA of 8 $Å^2$), with the last atom in the aliphatic tail most accessible (ASA of 6 $Å^2$). The aliphatic portion of this chain has a sharp kink in the middle, and ends up sandwiched between the aromatic ring of Phe141 and the branched-aliphatic Val132 side chain (*SI Appendix*, Figures S2A, S2B). The non-polar side chains that interact with this portion of the C3-acyl tail are all conserved in EcLpxH and KpLpxH, and would form the same or very similar interactions with the product, producing a roughly 120° bend in the aliphatic chain (*SI Appendix*, Figure S2). Of the water molecules completely buried in the binding site for the acyl chains, one is conserved in the inhibitor complex structures.

All of our inhibitors follow the path of the C3-acyl chain of the product from the glucosamine, with the L-shaped portion of the inhibitor at the central sulfonamide moiety causing a rapid change in direction (*SI Appendix*, Figure S3F), which mimics the bend seen for the product's aliphatic chain in the EcLpxH and KpLpxH structures (*SI Appendix*, Figure S2). Many of the interactions made in the product complex are also found with the inhibitors, with some notable exceptions. In all of the inhibitor complexes, the C-terminal end of the lid helix α6 mentioned above becomes disordered between residues 158-169, and the side chain of Arg80 adopts a new conformation (*SI Appendix*, Figures S2A, S2C). The beginning of the disorder varies slightly depending on the enzyme and complex; in the EcLpxH-/JEDI-1444 complex, for example, the disorder begins after Ser160. The first three residues following the disordered portion of the helix (170-172) take on a conformation in all inhibitor complexes that is different from that seen in the product complex. The side chain of Glu44 undergoes a small conformational change; it forms salt link/hydrogen bonding-interactions with Arg80 and Arg157 in the product complex, but accepts a hydrogen bond from ND1 of His10 in the inhibitor

complexes, as a result of a flip of the imidazole ring that coordinates one of the metal ions in the active site.

The inhibitors that we discuss here and in the main text all have a pair of rings on each side of the central sulfonamide group; the exception is JEDI-852, which lacks ring 1. The conformation of the sulfonamide group results in a rapid change in direction at this point. The side chains that interact with rings 1, 2 and the inward-directed face of ring 3 of the inhibitor are essentially unchanged in conformation when compared to the EcLpxH/product complex, whether in complex with EcLpxH or KpLpxH (*SI Appendix*, Figure S2). The cores of each inhibitor are, therefore, closely superimposable (*SI Appendix*, Figure S3F). The largest outlier is the piperazine ring of JEDI-852, which because it lacks the aromatic ring 1, is able to make a small re-orientation in ring 2 (*SI Appendix*, Figure S3C), which presumably optimizes the interaction with the enzyme.

Twenty-four residues show a change in ASA on the removal of the JEDI-1444 inhibitor from the model of the complex (a total change of 443 $Å^2$ for the enzyme). Two carbon atoms making up one edge of ring 1 are solvent accessible in the EcLpxH/JEDI-1444 complex (ASA of 4.5 and 19.2 $Å^2$, respectively), allowing a site for the addition of new groups. EBL-3339 and EBL-3647 both can accommodate a cyano-group at the more accessible site without disrupting binding. One carbon atom of the piperazine is barely solvent accessible (ASA 0.2 $Å^2$), and is close to the buried structurally-conserved water molecule observed in the product complex (closest approach 3.35 Å). The change in conformation of Arg80 allows the guanidino group to stack on one face of ring 3, a feature of all of the enzyme-inhibitor complexes. The connection between rings 3 and 4 is via a peptide linkage that runs in the opposite direction compared to the peptide-like linkage in the product. The respective peptide planes are only roughly co-planar (their plane normals make an angle of 51° when comparing the product and JEDI-1444 structures), but their respective carbonyl oxygen atoms each accept a hydrogen bond from the ND1 of Asn79. The flat aromatic ring 4 of our inhibitors is roughly co-planar with the chair conformation of the glucose ring of the product, but shifted ~3 Å, sliding over the ring of Tyr125 towards the ring of Phe128 (*SI Appendix*, Figure S2). The *ortho*-substituted sulfonamide of ring 4 overlaps with the phosphate group of the product, but only the repositioned Arg80 side chain interacts with the oxygens of the sulfonamide; the side chain of the second arginine involved in phosphate interactions with the product (Arg157) is poorly defined in all inhibitor complexes. Both methyl groups in the sulfonamide are solvent accessible and could be extended. The three carbon atoms at the *meta*- and *para*- positions of ring 4 are also accessible to solvent in the JEDI-1444, JEDI-852 and EBL-3339 complexes (ASA in the range 6-14 $Å^2$, in the JEDI-1444 complex), which allows the added group in EBL-3647 to be accepted without disrupting the binding of the core as a whole (*SI Appendix*, Figure S3F). The apparent openness of the binding site for ring 4 is, in part, a consequence of the disorder in $\alpha6$; we lack electron density and thus coordinates for 12 consecutive residues in our model, meaning that changes here need not compete with existing strong interactions. The meta-extension at the C5 atom in EBL-3647 follows the path of the C2-acyl chain, ending at the start of the aliphatic portion of the acyl chain (*SI Appendix*, Figure S2D). Larger extensions relative to the inhibitor core are, therefore, possible from ring 4, as well as from ring 1; changes at either could be designed to make specific interactions with the enzyme that could include following the path of the C2-acyl chain of the product. Of the seven residues that are involved in hydrogen-bonding or salt-link

interactions with the product, only two form hydrogen-bonding interactions with the inhibitors (residues 79 and 80); three are completely disordered (160, 164, 167), and one is partially disordered (Arg157’s side chain).

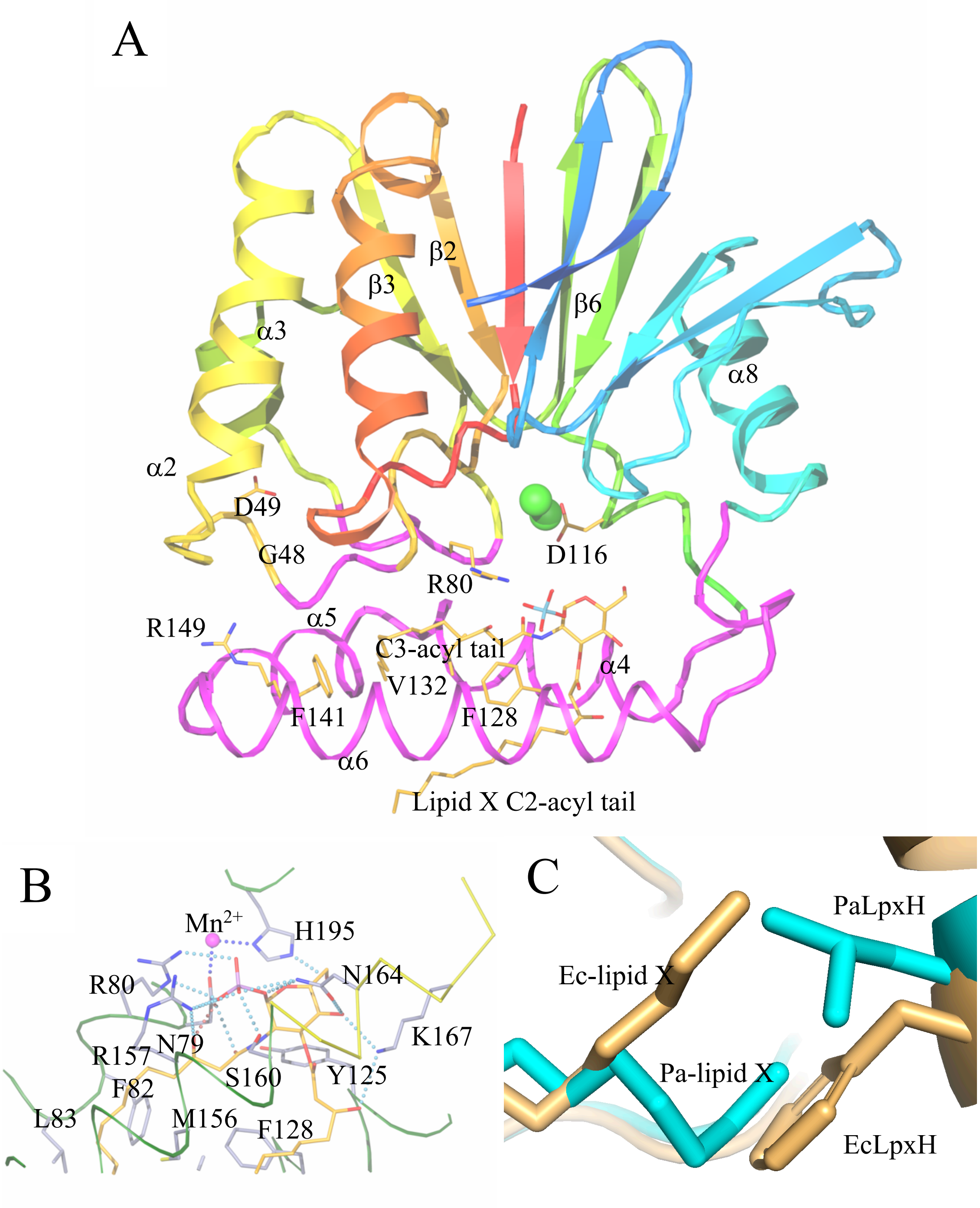

Figure S1. A. 3D cartoon of EcLpxH/lipid X (PDB code 8QJZ) with rainbow coloring (red to blue from N- to C-termini). Regions of the enzyme that interact with the product and/or inhibitors are colored magenta. Residues in LpxH identified in mutants selected against JEDI-852, AZ1, JEDI-1444, EBL-3599, and EBL-3647 (Tables S1 and S7) are indicated (carbon orange, nitrogen blue, oxygen red, sulfur yellow, phosphorus purple), as is the product. The metal ions at the active site are indicated as green spheres. **B.** The polar portion of the lipid X-binding site in the EcLpxH/product complex (PDB code 8QJZ). Atoms are drawn with atom-coloring (carbon silver,

nitrogen blue, oxygen red, sulfur yellow, phosphorus purple) except for the product where the carbons are drawn in orange. The glucosamine-1-P group of lipid X is locked in position by a complex set of interactions with enzyme side chains; sky blue bubbles indicate enzyme-product hydrogen bond/salt link interactions, dark blue bubbles indicate side chains that interact with one of the active-site manganese ions, and the orange bubbles indicate an intra-molecular hydrogen bond. The green portions of the cartoon indicate the main chain of the enzyme where there are significant enzyme-product interactions. The portion of the Cα-chain colored yellow is disordered in the EcLpxH/JEDI-1444 inhibitor complex. **C.** EcLpxH (gold, PDB code 8QJZ) and PaLpxH (cyan, (18)) product complexes in the vicinity of residue 141. The change in sequence Phe141Leu results in a modification to the product-binding site; a change in the conformation of the tip of the C3-acyl chain product is also apparent.

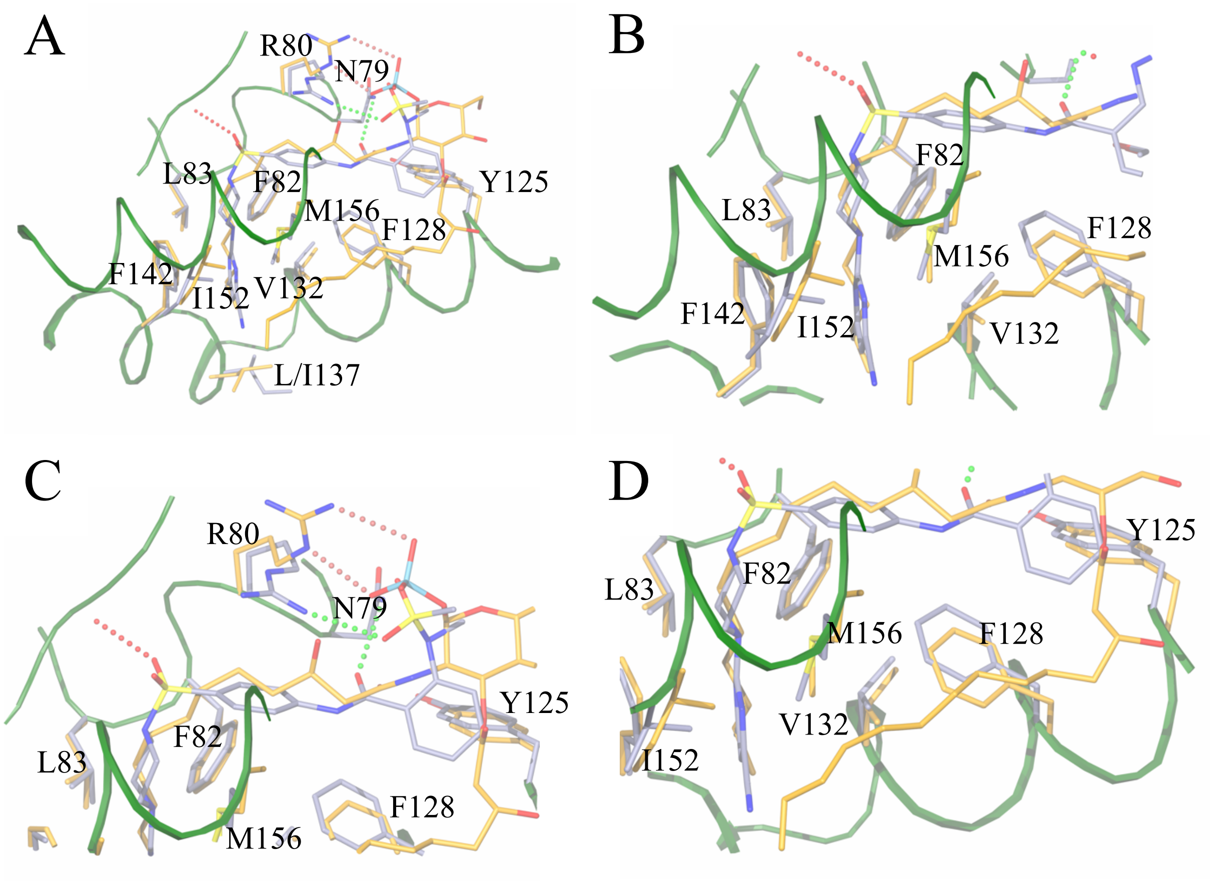

Figure S2. Superposition of the EcLpxH/lipid X (PDB code 8QJZ) and KpLpxH/EBL-3339 (PDB code 8QK2) binding sites. **A.** In an overview, atoms from the EcLpxH complex are colored orange, those of KpLpxH silver. Atoms are drawn with nitrogen blue, oxygen red, fluorine green, and sulfur yellow. For simplicity, the polar interactions involving the lipid X head group are omitted except those of Arg80 (orange bubbles), to emphasize the conformational change in this side chain. Polar interactions with JEDI-3339 are indicated by sky blue bubbles to enzyme side chains, and red to the main-chain nitrogen atom of residue 46. The side chains that interact with the acyl chains in the EcLpxH/product complex show only small conformational changes compared to the equivalent residues that interact with the inhibitor in the KpLpxH/EBL-3339 complex. The start of the disorder in the long α4 helix is a few residues earlier than in the EcLpxH/JEDI-1444 complex. Because of the complexity, panels B-D show the binding site in more detail, moving left to right in B and C, and down in D. Panel B highlights the interactions in the ring 1 and 2 sites, panel C in the ring 3 and 4 sites, and D the interacting residues from α4, together with the solvent-exposed acyl chain 2 of the product.

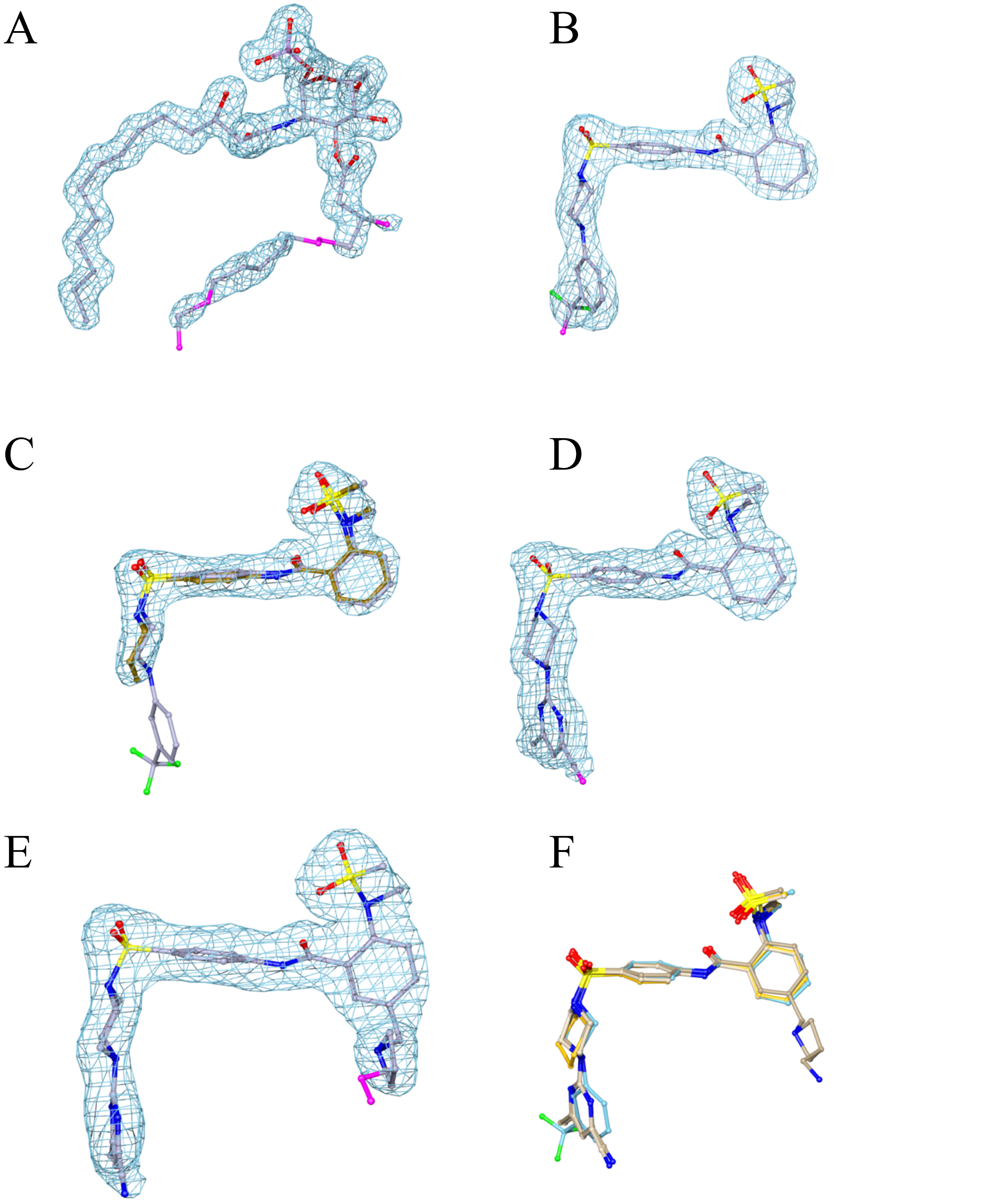

Figure S3. **Experimental electron density map around bound ligands (product or inhibitor) of LpxH.** Electron density is drawn at the RMS value of the relevant sigma$_A$-weighted (2m|Fo| -

D|Fc|) electron-density map (16) Carbon atoms differ in color as described. MEED outliers, bonds where some portion is not enveloped by electron density at the RMS of the map, are drawn in magenta. Other atoms are colored as in *SI Appendix*, Figure S2. **A.** Electron density around lipid X in the 1.35-Å resolution EcLpxH/lipid X complex (PDB code 8QJZ). The RMS value of the map is 0.355 e/Å^3. Carbon atoms are shown in silver; seven bonds are MEED outliers. The MEED outliers here are limited to portions of the 2-acyl chain where it reaches the external solvent. **B.** Electron density around JEDI-1444 in the 1.9 Å-resolution EcLpxH/inhibitor complex (PDB code 8QK9). The RMS value of the map is 0.225 e/Å^3. Carbon atoms are drawn in silver. There is a single MEED outlier. The three fluorine atoms lack significant bulges, and all come close to the envelope of the density, as indicated. **C.** Electron density around JEDI-852 in the 2.0-Å resolution KpLpxH/inhibitor complex (PDB code 8QKA). The RMS of the map is 0.23 e/Å^3. Carbon atoms are drawn in dark orange; there are no MEED outliers. The JEDI-1444 structure from the EcLpxH/inhibitor complex is superimposed after the alignment of the respective enzyme Cα atoms. JEDI-852 lacks the aromatic ring 1, which allows ring 2 to adopt a small conformational change that optimizes enzyme-inhibitor interactions. **D.** Electron density around EBL-3339 in the 1.95-Å resolution KpLpxH/inhibitor complex (PDB code 8QK2). The RMS of the map is 0.229 e/Å^3. Carbon atoms are drawn in silver; there is a single MEED outlier. The meta-substituted cyano group points towards solution, with the nitrogen atom close to the envelope of the density, as indicated. **E.** Electron density around EBL-3647 in the 1.95-Å resolution KpLpxH/inhibitor complex (PDB code 8QK5). The RMS of the map is 0.20 e/Å^3. Carbon atoms are drawn in silver; two bonds are MEED outliers. The C5-substituted group on ring 4 points towards solution, with two atoms at its tip outside the envelope of the density, as indicated. **F.** Superposition of the inhibitors from the four enzyme/inhibitor complexes; the alignments were made by the superpositioning of the respective Cα atoms onto the enzyme Cα coordinates of the EcLpxH/JEDI-1444 complex. Carbons from JEDI-1444 are shown in light blue, and those of JEDI-852 in orange, EBL-3339 in light brown, and EBL-3647 in darker brown.

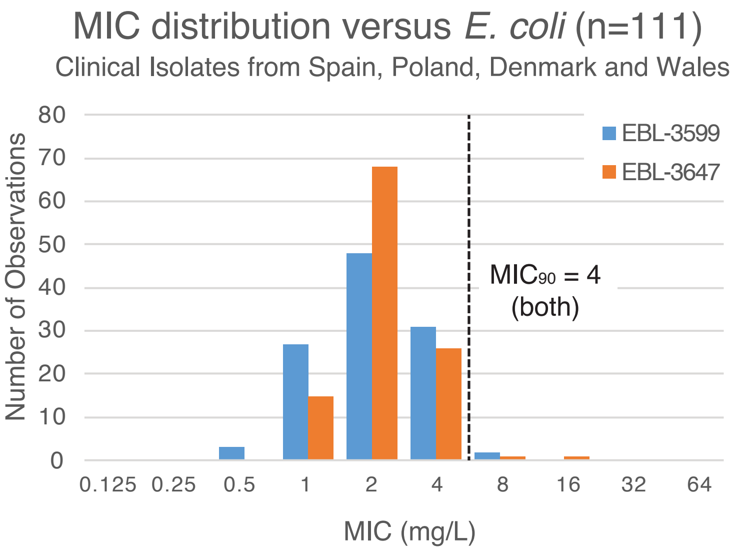

Figure S4. MIC distribution and MIC_{90} versus clinical *E. coli* isolates

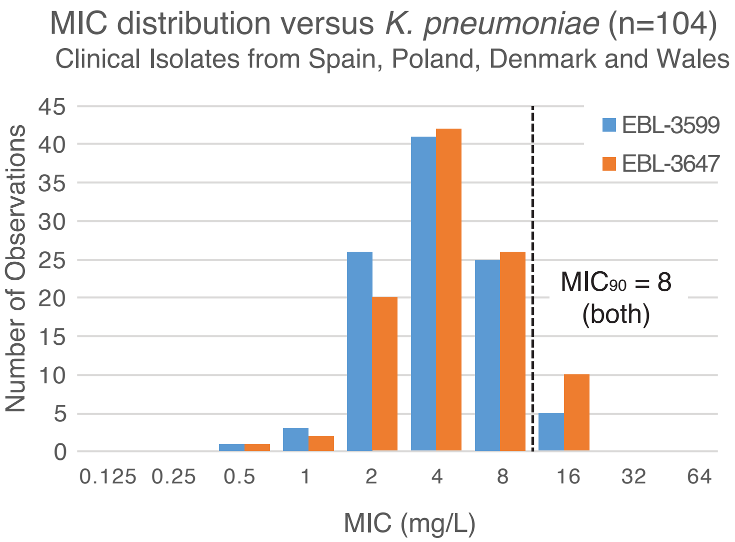

Figure S5. MIC distribution and MIC_{90} versus clinical *K. pneumoniae* isolates

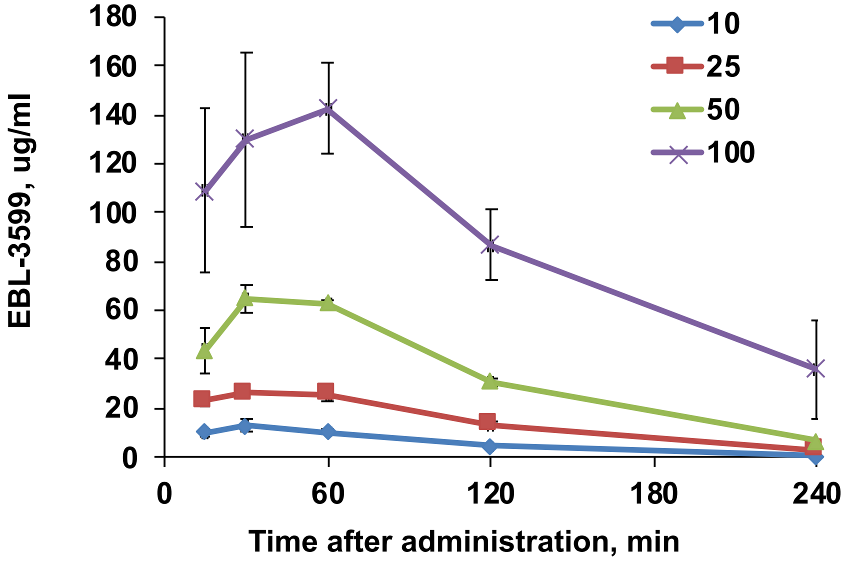

Figure S6. Concentrations of compounds in NMRI mice plasma after subcutaneous administration of EBL-3599 at doses of 10-100mg/kg. Bioanalysis of samples from each mouse and timepoint were done in duplicate and averaged. Data expressed as average ± S.D. of bioanalysis of samples from 3 mice.

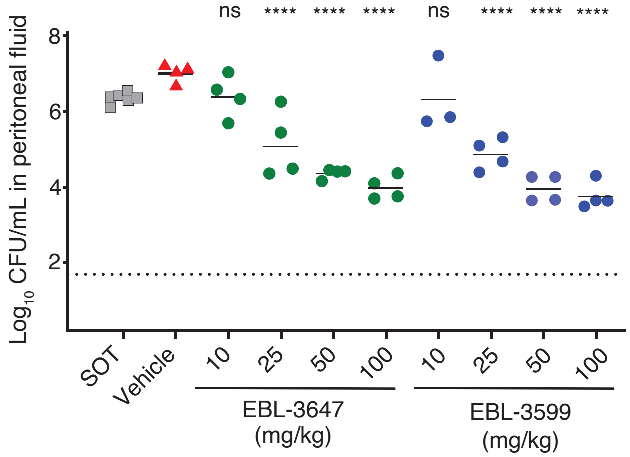

Figure S7. Bacterial cfu counts in peritoneal fluid for a dose-response *in vivo* efficacy study against *K. pneumoniae* (EN124) in a murine peritonitis model. Statistics for *in vivo* efficacy studies were calculated using a Dunnett's multiple comparisons test versus vehicle treatment: ns not significant, ****P<0.0001.

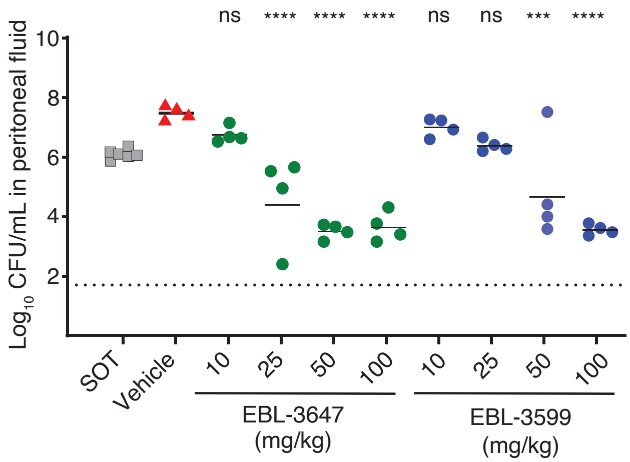

Figure S8. Bacterial cfu counts in peritoneal fluid for a dose-response *in vivo* efficacy study against *E. coli* (EN122) in a murine peritonitis model. Statistics for *in vivo* efficacy studies were calculated using a Dunnett's multiple comparisons test versus vehicle treatment: ns not significant, ***P<0.001, ****P<0.0001.

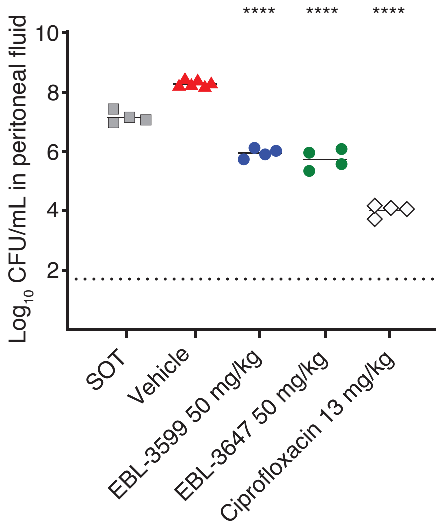

Figure S9. Bacterial cfu counts in peritoneal fluid for single-dose *in vivo* efficacy study against *K. pneumoniae* EN124 in a murine peritonitis model. Statistics for *in vivo* efficacy studies were calculated using a Dunnett's multiple comparisons test versus vehicle treatment: ns not significant, ****P<0.0001.

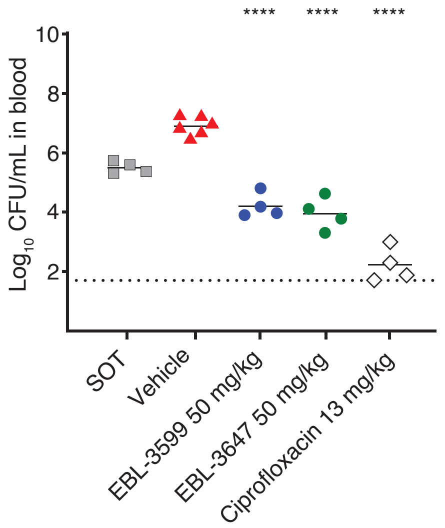

Figure S10. Bacterial cfu counts in blood for single-dose *in vivo* efficacy study against *K. pneumoniae* EN124 in a murine peritonitis model. Statistics for *in vivo* efficacy studies were calculated using a Dunnett's multiple comparisons test versus vehicle treatment: ns not significant, ****P<0.0001.

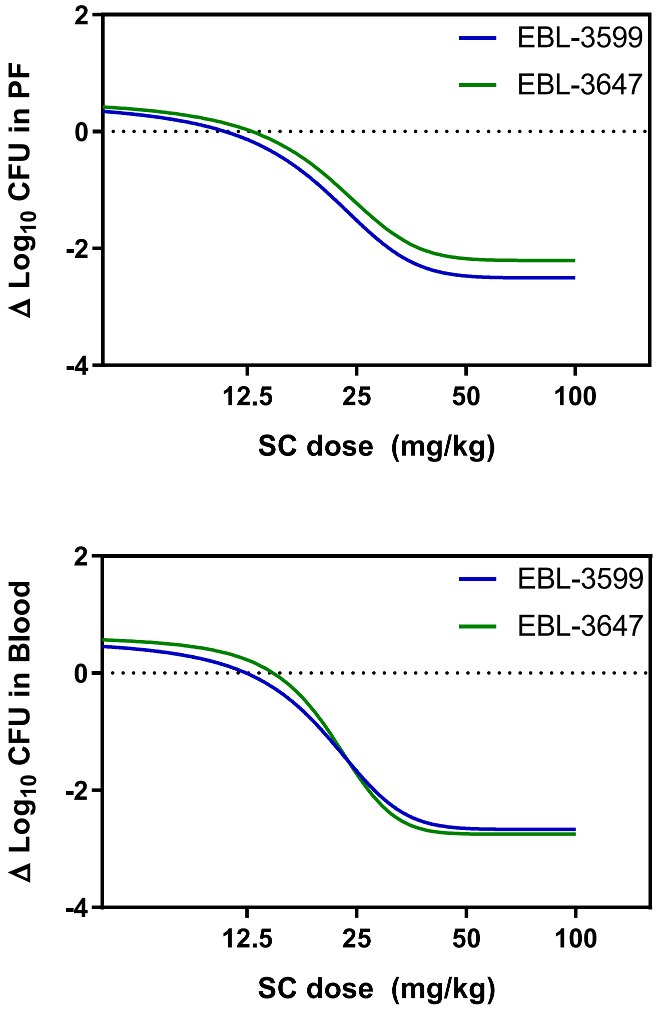

Figure S11. Modeled efficacy curves in peritoneal fluid (PF) and blood for EBL-3647 and EBL-3599 versus *K. pneumoniae* (EN124). Based on cfu counts from Figure 4D and *SI Appendix*, Figure S7.

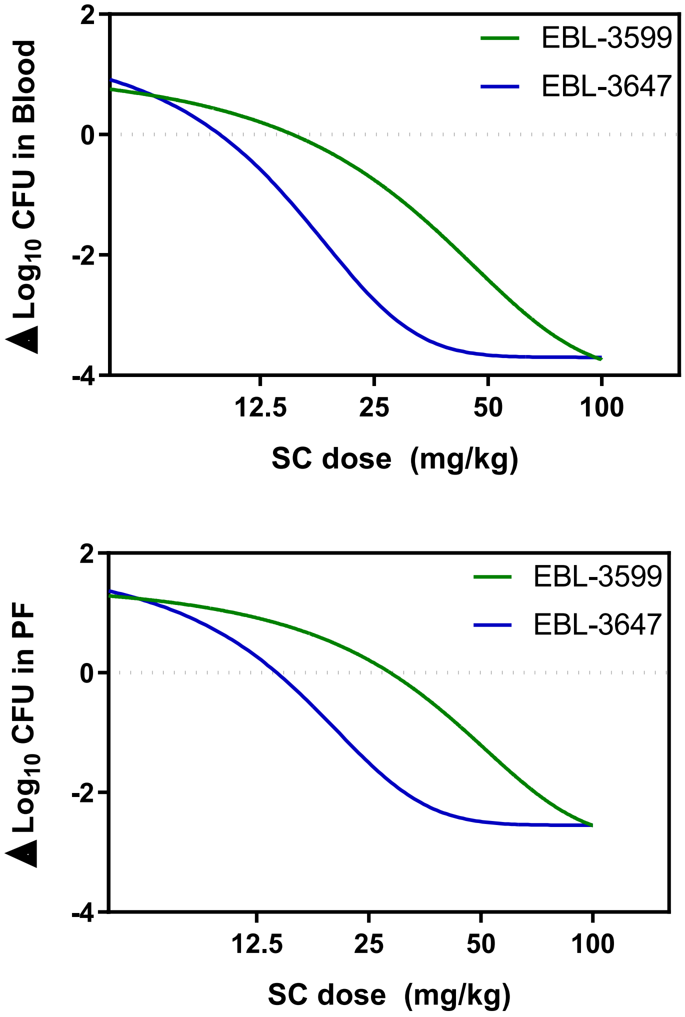

Figure S12. Modeled efficacy curves in peritoneal fluid (PF) and blood for EBL-3647 and EBL-3599 versus *E. coli* (EN122). Based on cfu counts from Figure 4C and *SI Appendix*, Figure S8.

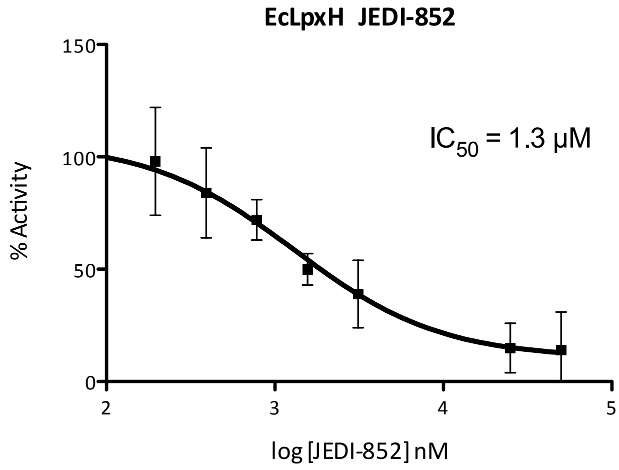

	Nonlin fit	% ACTIVITY
1	log(inhibitor) vs. response (three parameters)	
2	Best-fit values	
3	Bottom	10.43
4	Top	106.7
5	LogIC50	3.115
6	IC50	1304
7	Span	96.27
8	Std. Error	
9	Bottom	3.281
10	Top	2.952
11	LogIC50	0.07038
12	Span	3.986
13	95% Confidence Intervals	
14	Bottom	2.396 to 18.45
15	Top	99.47 to 113.9
16	LogIC50	2.943 to 3.288
17	IC50	877.4 to 1939
18	Span	86.52 to 106.0
19	Goodness of Fit	
20	Degrees of Freedom	6
21	R square	0.9898
22	Absolute Sum of Squares	105.1
23	Sy.x	4.184
24	Number of points	
25	Analyzed	9

Figure S13. IC_{50} curves and statistics for enzyme inhibition of *E. coli* LpxH by JEDI-852. EcLpxH concentration 0.1 nM, substrate concentration 20 μM.

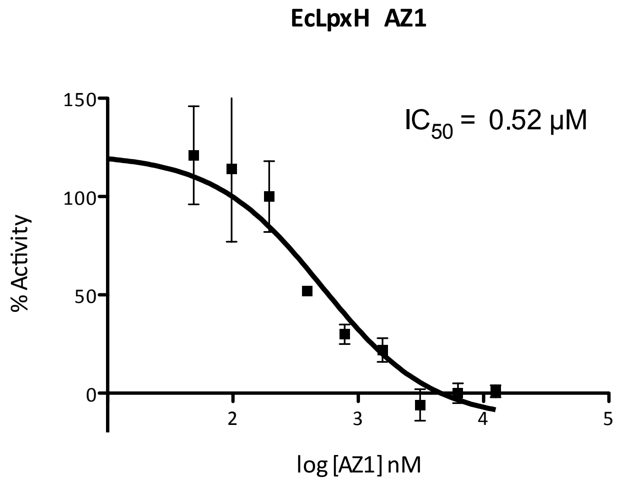

	Nonlin fit	A<br>% ACTIVITY
1	log(inhibitor) vs. response (three parameters)	
2	Best-fit values	
3	Bottom	-13.78
4	Top	121.8
5	LogIC50	2.714
6	IC50	517.8
7	Span	135.6
8	Std. Error	
9	Bottom	11.30
10	Top	10.87
11	LogIC50	0.1839
12	Span	13.38
13	95% Confidence Intervals	
14	Bottom	-40.50 to 12.94
15	Top	96.10 to 147.5
16	LogIC50	2.279 to 3.149
17	IC50	190.2 to 1409
18	Span	103.9 to 167.2
19	Goodness of Fit	
20	Degrees of Freedom	7
21	R square	0.9364
22	Absolute Sum of Squares	1479
23	Sy.x	14.54
24	Number of points	
25	Analyzed	10

Figure S14. IC_{50} curves and statistics for enzyme inhibition of *E. coli* LpxH by AZ1. EcLpxH concentration 0.1 nM, substrate concentration 20 μM.

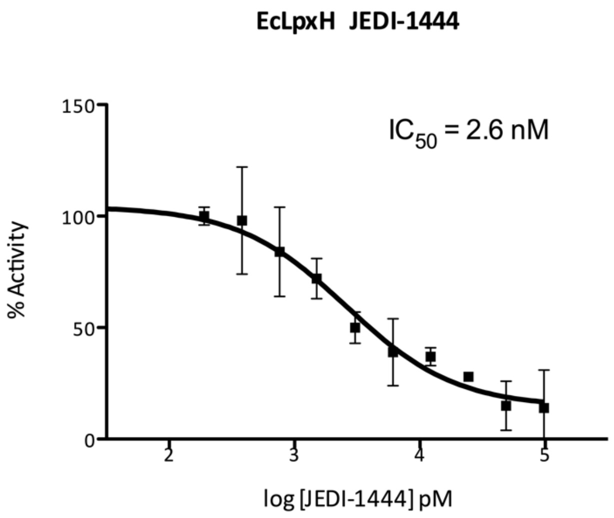

	Nonlin fit	A<br>% ACTIVITY
1	log(inhibitor) vs. response (three parameters)	
2	Best-fit values	
3	Bottom	14.30
4	Top	104.4
5	LogIC50	3.417
6	IC50	2614
7	Span	90.08
8	Std. Error	
9	Bottom	3.225
10	Top	3.319
11	LogIC50	0.08462
12	Span	4.018
13	95% Confidence Intervals	
14	Bottom	6.859 to 21.73
15	Top	96.72 to 112.0
16	LogIC50	3.222 to 3.612
17	IC50	1668 to 4097
18	Span	80.81 to 99.34
19	Goodness of Fit	
20	Degrees of Freedom	8
21	R square	0.9843
22	Absolute Sum of Squares	181.0
23	Sy.x	4.756
24	Number of points	
25	Analyzed	11

Figure S15. IC_{50} curves and statistics for enzyme inhibition of *E. coli* LpxH by JEDI-1444. EcLpxH concentration 0.1 nM, substrate concentration 20 μM.

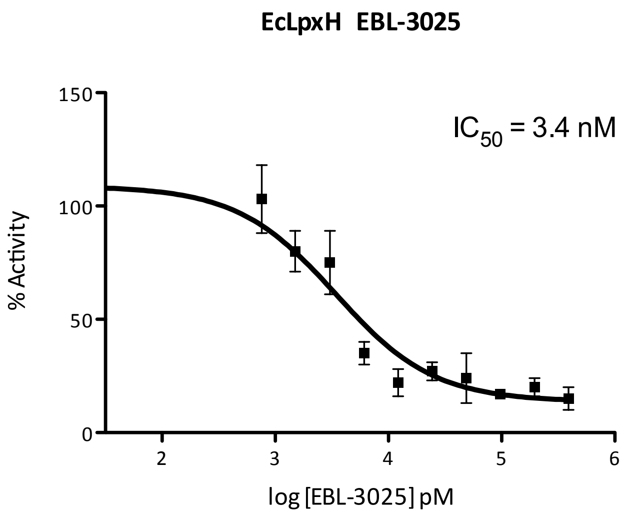

	Nonlin fit	A<br>% ACTIVITY
1	log(inhibitor) vs. response (three parameters)	
2	Best-fit values	
3	Bottom	13.68
4	Top	108.8
5	LogIC50	3.533
6	IC50	3410
7	Span	95.12
8	Std. Error	
9	Bottom	4.891
10	Top	8.163
11	LogIC50	0.1503
12	Span	8.711
13	95% Confidence Intervals	
14	Bottom	2.402 to 24.96
15	Top	89.97 to 127.6
16	LogIC50	3.186 to 3.879
17	IC50	1535 to 7575
18	Span	75.03 to 115.2
19	Goodness of Fit	
20	Degrees of Freedom	8
21	R square	0.9429
22	Absolute Sum of Squares	695.0
23	Sy.x	9.320
24	Number of points	
25	Analyzed	11

Figure S16. IC_{50} curves and statistics for enzyme inhibition of *E. coli* LpxH by EBL-3025. EcLpxH concentration 0.1 nM, substrate concentration 20 μM.

.

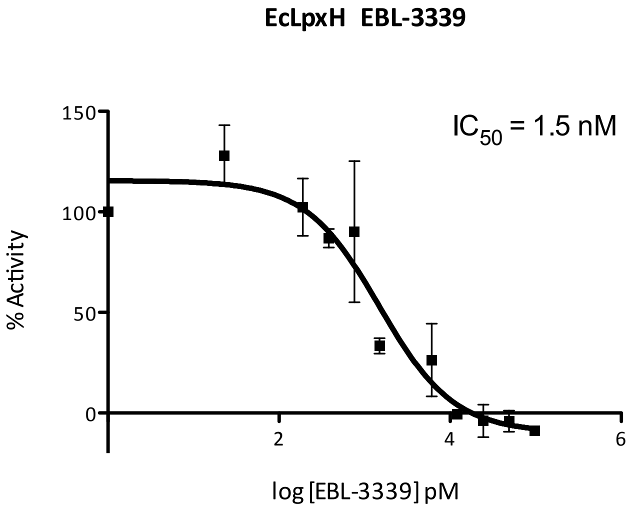

	Nonlin fit	A<br>% ACTIVITY
1	log(inhibitor) vs. response (three parameters)	
2	Best-fit values	
3	Bottom	-9.635
4	Top	115.6
5	LogIC50	3.174
6	IC50	1494
7	Span	125.2
8	Std. Error	
9	Bottom	7.382
10	Top	8.114
11	LogIC50	0.1586
12	Span	9.880
13	95% Confidence Intervals	
14	Bottom	-26.66 to 7.389
15	Top	96.85 to 134.3
16	LogIC50	2.808 to 3.540
17	IC50	643.3 to 3468
18	Span	102.4 to 148.0
19	Goodness of Fit	
20	Degrees of Freedom	8
21	R square	0.9528
22	Absolute Sum of Squares	1273
23	Sy.x	12.62
24	Number of points	
25	Analyzed	11

Figure S17. IC_{50} curves and statistics for enzyme inhibition of *E. coli* LpxH by EBL-3339. EcLpxH concentration 0.1 nM, substrate concentration 20 μM.

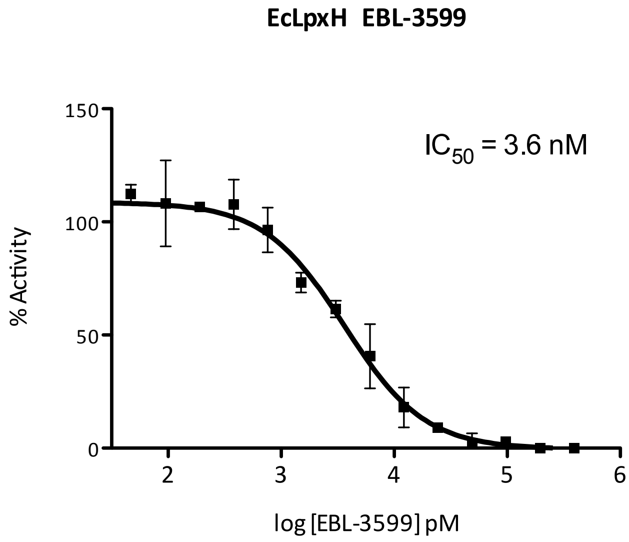

	Nonlin fit	% ACTIVITY
1	log(inhibitor) vs. response -- Variable slope (four para	
2	Best-fit values	
3	Bottom	-0.4999
4	Top	108.7
5	LogIC50	3.559
6	HillSlope	-1.221
7	IC50	3622
8	Span	109.2
9	Std. Error	
10	Bottom	2.581
11	Top	2.378
12	LogIC50	0.04428
13	HillSlope	0.1374
14	Span	3.863
15	95% Confidence Intervals	
16	Bottom	-6.180 to 5.180
17	Top	103.5 to 113.9
18	LogIC50	3.461 to 3.656
19	HillSlope	-1.524 to -0.9191
20	IC50	2893 to 4533
21	Span	100.7 to 117.7
22	Goodness of Fit	
23	Degrees of Freedom	11
24	R square	0.9931
25	Absolute Sum of Squares	211.7
26	Sy.x	4.387
27	Number of points	
28	Analyzed	15

Figure S18. IC_{50} curves and statistics for enzyme inhibition of *E. coli* LpxH by EBL-3599. EcLpxH concentration 0.1 nM, substrate concentration 20 μM.

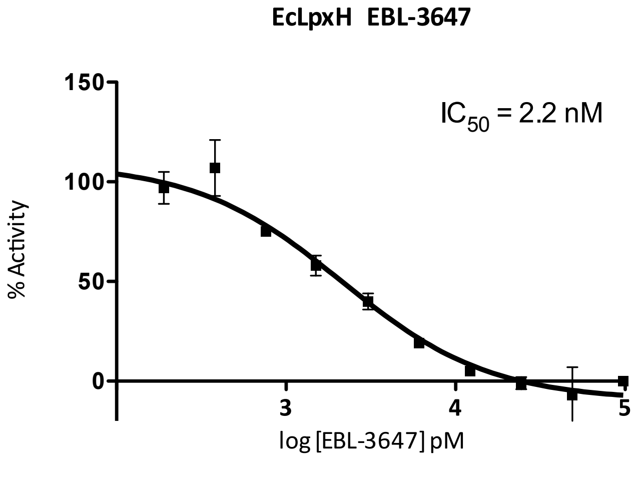

	Nonlin fit	A<br>% ACTIVITY
1	log(inhibitor) vs. response (three parameters)	
2	Best-fit values	
3	Bottom	-9.603
4	Top	109.2
5	LogIC50	3.332
6	IC50	2149
7	Span	118.8
8	Std. Error	
9	Bottom	4.442
10	Top	4.320
11	LogIC50	0.08892
12	Span	5.441
13	95% Confidence Intervals	
14	Bottom	-19.65 to 0.4447
15	Top	99.42 to 119.0
16	LogIC50	3.131 to 3.533
17	IC50	1352 to 3415
18	Span	106.5 to 131.1
19	Goodness of Fit	
20	Degrees of Freedom	9
21	R square	0.9815
22	Absolute Sum of Squares	431.9
23	Sy.x	6.928
24	Number of points	
25	Analyzed	12

Figure S19. IC_{50} curves and statistics for enzyme inhibition of *E. coli* LpxH by EBL-3647. EcLpxH concentration 0.1 nM, substrate concentration 20 μM.

Supplementary Tables.

Table S1. LpxH mutants with reduced susceptibility in efflux-defective *E. coli* background.

Strain	Parental Strain	Compound Selected Against	Mutation	MIC (mg/L)		
				JEDI-852	AZ1	JEDI-1444
EN243	*Eco* MG1655	-	*ΔtolC*	4	0.125	0.016
CH6045	EN243	JEDI-852	*lpxH* G48D	64	2	0.25
CH6047	EN243	JEDI-852	*lpxH* F128L	> 64	> 64	1
CH6048	EN243	JEDI-852	*lpxH* D116N	32	0.5	0.064
CH6051	EN243	JEDI-852	*lpxH* F141L	> 64	> 64	> 2
CH7010	EN243	AZ1	*lpxH* V132A	2	2	0.064
CH7011	EN243	AZ1	*lpxH* G48C	16	2	0.25
CH7012	EN243	AZ1	*lpxH* D49Y	16	4	0.064
CH7013	EN243	AZ1	*lpxH* ~25-fold Amplification	> 64	64	0.125
CH7015	EN243	AZ1	*lpxH* D49Y	32	> 64	0.064
CH7016	EN243	AZ1	*lpxH* D49Y	16	2	0.125
CH8845	EN243	JEDI-1444	*lpxH* F128L	32	2	1
CH8846	EN243	JEDI-1444	*lpxH* G48D	32	2	0.25
CH8847	EN243	JEDI-1444	*lpxH* F141L	> 64	> 64	> 2
CH8848	EN243	JEDI-1444	*lpxH* R80H	> 64	4	0.5
CH8850	EN243	JEDI-1444	*lpxH* R80H	> 64	2	0.5
CH8851	EN243	JEDI-1444	*lpxH* G48S	8	1	0.25
CH8853	EN243	JEDI-1444	*lpxH* R80H	> 64	2	0.25
CH8854	EN243	JEDI-1444	*lpxH* R80H	> 64	2	0.25
CH8855	EN243	JEDI-1444	*lpxH* F141L	> 64	> 64	> 2
CH8856	EN243	JEDI-1444	*lpxH* F141L	> 64	> 64	> 2
CH8868	EN243	JEDI-1444	*lpxH* F141S	> 64	> 64	2
CH8869	EN243	JEDI-1444	*lpxH* R80H	4	0.125	0.064
CH8870	EN243	JEDI-1444	*lpxH* R149L	32	> 64	0.25
CH8871	EN243	JEDI-1444	*lpxH F*141V	> 64	> 64	2

Table S2. X-ray data collection and refinement statistics.

Structure	EcLpxH/lipid X	EcLpxH/JEDI-1444	KpLpxH/JEDI-852	KpLpxH/EBL-3339	KpLpxH/EBL-3647
**PDB code**	8QJZ	8QK9	8QKA	8QK2	8QK5
**Environment**	IO4-1 (Diamond)	Biomax IV	Biomax IV	Diamond io3 (Diamond)	ESRF_id30a
**Wavelength**	0.91589	0.918401	0.918401	0.97625	0.965459
**Cell dimensions (Å)**	123.83 81.39 33.13 90.00 100.32 90.00	122.31 81.18 32.97 90.00 103.00 90.00	60.01 60.01 284.66 90.00 90.00 120	60.20 60.20 286.78 90.00 90.00 120.00	60.42 60.42 287.69 90.00 90.00 120.00
**Space group**	C 1 2 1	C 1 2 1	P 61 2 2	P 61 2 2	P 61 2 2
**Resolution (Å)**	67.66 - 1.35 (1.42 - 1.35)	67.06 - 1.9 (2.0 -1.9)	29.36 - 2.0 (2.05 - 2.0)	29.94 -1-95 (2.0 - 1.95)	29.64 - 1.95 (2.00 - 1.95)
**Unique reflections**	70366	24774	21782	21114	23964
**Average multiplicity**	6.1 (4.4)	7.1 (7.1)	36.0 (34.0)	20.7 (21.8)	7.7 (7.9)
**Completeness (%)**	99.4 (96.2)	100 (100.0)	100 (100.0)	88.5 (100.0)	99.9 (100.0)
$\mathbf{R_{merge}}$	7.0 (68.0)	9.1 (81.5)	10.9 (75.2)	9.0 (16.7)	5.5 (79.2)
$\mathbf{R_{mean}}$	0.076 (0.775)	0.098 (0.880)	0.112 (0.773)	0.094 (1.747)	0.061 (0.885)
**R.p.i.m**	0.030 (0.360)	0.037 (0.329)	0.024 (0.177)	0.028 (0.507)	0.025 (0.374)
**<I/sd(I)>**	11.1 (1.5)	10.7 (2.1)	18.2 (5.4)	20.5 (2.2)	12.5 (2.0)
**Aniso <I/sd(I)> [a] (resolution)**	1.1, 1.7, 4.8 (1.35-1.42)	1.0, 1.3, 7.2 (1.90-2.00)	3.0, 2.4, 3.7 (2.0-2.11)	2.2, 2.2, 2.5 (1.95-2.05)	1.3, 1.3, 4.1 (1.95-2.05)

**Wilson plot B factor**	12.6	31.7	31.1	36.1	44.2
**CC 1/2**	0.999 (0.827)	0.999 (0.814)	0.999 (0.955)	0.999 (0.792)	0.999 (0.652)
**Refinement**					
**Number of reflections (completeness , %)**	66887	23478	20615	19918	22624
**Resolution range (Å)**	1.35	1.90	2.0	1.95	1.95
**R-factor / R-free (%)**	17.3 / 18.8	17.9 / 21.2	22.7 / 26.6	22.6 / 25.4	24.0 / 28.3
**Number of protein atoms**	1892	1829	1836	1850	1844
**Number of water molecules**	233	74	43	59	48
**Number of ligand atoms**	48	40	30	39	46
**Mn^{2+}**	2	2	2	2	2
**Average B (inhibitor) , Å^2**	19.5 (27.3)	41.1 (50.8)	41.6 (42.7)	42.3 (38.5)	50.9 (50.2)
**r.m.s bond length (Å)**	0.007	0.010	0.006	0.007	0.008
**r.m.s. bond angle (°)**	1.27	1.53	1.17	1.18	1.34
**Ramachandran plot outliers (number, %)[b]**	0	0	0	0	0

Statistics for data collection and refinement were generated by computer programs referenced in the Methods section, unless otherwise stated:

[a]The mean (I(hkl)/standard-deviation(I(hkl)) is evaluated in the highest resolution range along the orthogonal axes of anisotropy using the HKL-tools in O.

[b]PDB validation report.

Table S3. MICs of EBL-3599 and EBL-3647 against various species of laboratory strains

Strain Number	Species	Genotype/Phenotype	Source/ Reference	MIC (mg/L)	
				**EBL-3599**	**EBL-3647**
ATCC 29213	*S. aureus*	WT	ATCC	>64	>64
BM4454	*A. baumanii*	WT	(20)	> 64	> 64
BM4652	*A. baumanii*	Efflux-defective BM4454	(21)	32	64
PAO1	*P. aeruginosa*	WT	(22)	> 64	> 64
PAO750	*P. aeruginosa*	Efflux-defective PAO1	(23)	64	64
ATCC 7002	*P. mirabilis*	WT	ATCC	> 64	> 64
EN1490	*P. mirabilis*	*tolC*::Tn5-kan2 ATCC 7002	This work	4	16

Table S4. MICs of EBL-3599 and EBL-3647 against clinical multi-drug resistant (MDR) *E. coli* and *K. pneumoniae* isolates

Strain Number	Species	Genotype/Phenotype	MIC (mg/L)					
			**EBL-3599**	**EBL-3647**	**Meropenem**	**Gentamicin**	**Colistin**	**Cefotaxime**
EN0001	*E. coli*	**ATCC 25922**	2	2	≤ 0.125	1	2	0.5
EN0134	*E. coli*	**MDR** (aadA5, aph(3'')-Ib, aph(6)-Id, blaCMY-4, blaTEM-1B, qacE, sitABCD, sul1, sul2, dfrA17)	2	2	≤ 0.125	2	2	8
EN0135	*E. coli*	**MDR** (aac(3)-IIa, blaTEM-1B, blaSHV-12, sitABCD, catA1, tet(A))	2	2	≤ 0.125	> 64	2	2
EN0136	*E. coli*	**MDR** (aac(3)-IIa, aac(6')-Ib3,aadA5, blaOXA-2, blaTEM-1B, blaCTX-M-2, qacE, sitABCD, sul1, sul2, dfrA17)	2	4	≤ 0.125	> 64	1	4
EN0137	*E. coli*	**MDR** (aac(3)-IIa, aadA5, blaOXA-1, blaTEM-1B, blaCTX-M-15, qacE, sitABCD, aac(6')-Ib-cr, mph(A), catB3, sul1, dfrA17)	4	2	≤ 0.125	> 64	2	>64
EN0138	*E. coli*	**MDR** (aadA1, ant(2'')-Ia, aph(3'')-Ib, aph(6)-Id, blaCTX-M-14b, blaTEM-1C, qacE, sitABCD, mph(A), cmlA1, sul1, sul2, tet(B), dfrA24)	2	4	≤ 0.125	8	2	>64
EN0139	*E. coli*	**MDR** (aadA5, blaOXA-1, blaCTX-M-15, qacE, sitABCD, aac(6')-Ib-cr, mph(A), catA1, catB3, sul1, tet(B), dfrA17)	4	4	≤ 0.125	2	2	>64
EN0140	*K. pneumoniae*	**MDR** (aac(3)-IIa, aadA2, aph(3')-Ia, blaOXA-1, blaSHV-182, blaTEM-1B, blaDHA-1, OqxA, OqxB, qacE, aac(6')-Ib-cr, fosA, mph(A), catA1, catB3, qnrB4, ARR-3, sul1, sul2, dfrA12), OmpK36 loss	1	1	2	> 64	2	>64

EN0233	*K. pneumoniae*	**carbapenemR (**blaOXA-48), **MDR** (aac(3)-IIa, aph(3'')-Ib, aph(6)-Id, blaOXA-1, blaSHV-76, blaTEM-1B, blaCTX-M-15, OqxA, OqxB, aac(6')-Ib-cr, fosA, catB3, qnrB1, sul2, tet(A), dfrA14), OmpK36 loss	4	4	2	> 64	32	>64
EN0142	*K. pneumoniae*	**carbapenemR (**blaKPC-2), **MDR** (aac(6')-Ib, aadA2, aph(3')-Ia, blaOXA-9, blaSHV-182, blaTEM-1A, OqxA, OqxB, qacE, fosA, mph(A), catA1, sul1, dfrA12)	2	2	0.5	64	2	>64
EN0143	*K. pneumoniae*	**MDR** (aac(6')-Il, aadA1b, blaOXA-2, blaSHV-12, qacE, OqxA, OqxB, fosA, sul1, dfrA29)	2	2	≤ 0.125	0.5	2	16
EN0144	*K. pneumoniae*	**MBL (**blaVIM-26), **MDR** (aac(6')-Il, aadA23, aph(2'')-Ib, aac(6')-Im, aph(3')-Ia, aph(3'')-Ib, aph(6)-Id, blaSHV-165, qacE, OqxB, OqxA, fosA, mph(A), sul1, dfrA1)	2	2	64	> 64	2	>64
EN0145	*K. pneumoniae*	**MDR** (aac(3)-IIa, blaOXA-1, blaSHV-182, blaTEM-1B, blaCTX-M-15, OqxA, OqxB, aac(6')-Ib-cr, fosA, catB3)	4	4	8	2	2	>64
EN0303	*E. coli*	**colistinR** (pmrB T156M(24)), **MDR** (aadA2, aph(6)-Id, blaCTX-M-14, blaTEM-1B, , cmlA1, sul2, sul3, tet(A), dfrA14)	16	16	≤ 0.125	2	8	>64
EN0481	*E. coli*	**colistinR** (mcr-1), **MDR** (aadA5, rmtB, strA, strB, blaCMY-2, blaCTX-M-55, blaTEM-1B, catA1, dfrA17, floR, fosA, mph(A), sul1, sul2, tet(A))	2	1	≤ 0.125	> 64	8	>64
EN0245	*K. pneumoniae*	**colistinR** (mgrB::IS10 (25)), **MDR** (aadA17, aac(6')-Ib, aadA1, blaSHV-11, blaTEM-1A, blaOXA-9, blaDHA-1, fosA, qnrB4, catA1, oqxA, oqxB, sul1, dfrA12)	8	4	2	2	64	64
EN0504	*K. pneumoniae*	**colistinR**, **MDR** (aadA2, blaSHV-182, blaCTX-M-15, blaDHA-1, OqxB, OqxA, qacE, fosA, catA1, qnrB4, sul1, dfrA12)	4	4	4	0.5	64	>64

Table S5. Frequency of resistance (FoR) at 24 hours, at 4 times measured agar MIC

Compound		*E. coli* ATCC 25922	*K. pneumoniae* ATCC 13883
EBL-3599	Agar MIC (mg/L)	2	0.5
	FoR Conc. (mg/L)	8	2
	FoR	$< 8.5 \times 10^{-10}$	1.6×10^{-9}
EBL-3647	Agar MIC (mg/L)	2	0.5
	FoR Conc. (mg/L)	8	2
	FoR	$< 8.5 \times 10^{-10}$	5.4×10^{-10}

Table S6. Mutant classes from frequency of resistance experiment

		Total Mutant Isolates Sequenced	*lpxH* mutants	Other mutants
EBL-3599	*E. coli* ATCC 25922	0	-	-
	K. pneumoniae ATCC 13883	3	3	0
EBL-3647	*E. coli* ATCC 25922	0	-	-
	K. pneumoniae ATCC 13883	4	3	1 (*ramR*)

Table S7. Complete identity and MICs of mutants from frequency of resistance experiments

**Strain**	**Parental**	**Selected against:**	**EBL-3599 MIC**	**EBL-3647 MIC**	**lpxH genotype**	**other mutations**
EN0006	ATCC 13883	Parent	1	1	wild-type	
CH10205	EN0006	EBL-3599	32	32	*lpxH* R80H	
CH10206	EN0006	EBL-3599	> 64	> 64	*lpxH* F141S	
CH10207	EN0006	EBL-3599	32	32	*lpxH* R80H	
CH10218	EN0006	EBL-3647	64	32	*lpxH* F128L	
CH10219	EN0006	EBL-3647	8	8	wild-type	*ramR* A40V
CH10220	EN0006	EBL-3647	> 64	> 64	*lpxH* F141S	
CH10221	EN0006	EBL-3647	> 64	> 64	*lpxH* R80H	

Table S8. Effective doses (mg/kg) against *K. pneumoniae* peritoneal infection calculated from modeled efficacy curves (*SI Appendix*, Figure S11).

	Peritoneal Fluid		Blood	
	EBL-3647	EBL-3599	EBL-3647	EBL-3599
ED_{50}	21	19.78	21	20.2
1-log	22.8	20.4	21	20.2
2-log	37.3	30.7	27	28

Table S9. Effective doses (mg/kg) against E. coli peritoneal infection calculated from modeled efficacy curves (*SI Appendix*, Figure S12).

	Peritoneal Fluid		Blood	
	EBL-3647	EBL-3599	EBL-3647	EBL-3599
ED_{50}	13.6	29.6	12.3	20.0
1-log	20.8	45.9	14.5	28.3
2-log	31.2	70.0	19.6	42.8
3-log			27.6	63.4

Table S10. Calculated PK parameters in NMRI mice plasma after subcutaneous administration of EBL-3647 at doses of 10-100mg/kg. Data expressed as average from 3 mice. Corresponding plasma concentrations for calculations presented in main text in Figure 4, panel B.

Dose (mg/kg):	10	25	50	100
C_{max} (µg/mL)	12	30	72	130
AUC (µg/mL*min)	1215	3315	8378	13313

Table S11. Calculated PK parameters in NMRI mice plasma after subcutaneous administration of EBL-3599 at doses of 10-100mg/kg. Data expressed as average from 3 mice. Corresponding plasma concentrations for calculations presented in *SI Appendix*, Figure S6.

Dose (mg/kg):	10	25	50	100
C_{max} (µg/mL)	13	27	65	142
AUC (µg/mL*min)	1373	3479	8150	20934

Supplementary References

1. M. R. Wilkins, *et al.*, “Protein Identification and Analysis Tools in the ExPASy Server” in *2-D Proteome Analysis Protocols*, (Humana Press), pp. 531–552.
2. J. Zhao, *et al.*, The Lipid A 1-Phosphatase, LpxE, Functionally Connects Multiple Layers of Bacterial Envelope Biogenesis. *mBio* **10** (2019).
3. M. Lee, *et al.*, Structure–Activity Relationship of Sulfonyl Piperazine LpxH Inhibitors Analyzed by an LpxE-Coupled Malachite Green Assay. *ACS Infect Dis* **5**, 641–651 (2019).
4. F. Gorrec, The MORPHEUS protein crystallization screen. *J Appl Crystallogr* **42**, 1035–1042 (2009).
5. W. Kabsch, *XDS*. *Acta Crystallogr D Biol Crystallogr* **66**, 125–132 (2010).
6. P. R. Evans, G. N. Murshudov, How good are my data and what is the resolution? *Acta Crystallogr D Biol Crystallogr* **69**, 1204–1214 (2013).
7. A. J. McCoy, *et al.*, *Phaser* crystallographic software. *J Appl Crystallogr* **40**, 658–674 (2007).
8. G. N. Murshudov, *et al.*, *REFMAC* 5 for the refinement of macromolecular crystal structures. *Acta Crystallogr D Biol Crystallogr* **67**, 355–367 (2011).
9. T. A. Jones, J. Y. Zou, S. W. Cowan, M. Kjeldgaard, Improved methods for building protein models in electron density maps and the location of errors in these models. *Acta Crystallogr A* **47**, 110–119 (1991).
10. E. Lindhagen, P. Nygren, R. Larsson, The fluorometric microculture cytotoxicity assay. *Nat Protoc* **3**, 1364–1369 (2008).
11. M. Durcik, *et al.*, New Dual Inhibitors of Bacterial Topoisomerases with Broad-Spectrum Antibacterial Activity and In Vivo Efficacy against Vancomycin-Intermediate *Staphylococcus aureus*. *J Med Chem* **66**, 3968–3994 (2023).
12. V. Bortolaia, *et al.*, ResFinder 4.0 for predictions of phenotypes from genotypes. *Journal of Antimicrobial Chemotherapy* **75**, 3491–3500 (2020).
13. E. Zankari, *et al.*, PointFinder: a novel web tool for WGS-based detection of antimicrobial resistance associated with chromosomal point mutations in bacterial pathogens. *Journal of Antimicrobial Chemotherapy* **72**, 2764–2768 (2017).
14. C. Camacho, *et al.*, BLAST+: architecture and applications. *BMC Bioinformatics* **10**, 421 (2009).
15. G. L. Bundy, *et al.*, Heterocycle Carboxamides as Antiviral Agents (2002).
16. R. J. Read, Improved Fourier coefficients for maps using phases from partial structures with errors. *Acta Crystallogr A* **42**, 140–149 (1986).
17. J. Cho, C.-J. Lee, J. Zhao, H. E. Young, P. Zhou, Structure of the essential Haemophilus influenzae UDP-diacylglucosamine pyrophosphohydrolase LpxH in lipid A biosynthesis. *Nat Microbiol* **1**, 16154 (2016).
18. C. Okada, *et al.*, Crystal structures of the UDP-diacylglucosamine pyrophosphohydrase LpxH from Pseudomonas aeruginosa. *Sci Rep* **6**, 32822 (2016).
19. B. Lee, F. M. Richards, The interpretation of protein structures: Estimation of static accessibility. *J Mol Biol* **55**, 379-IN4 (1971).
20. S. Magnet, P. Courvalin, T. Lambert, Resistance-Nodulation-Cell Division-Type Efflux Pump Involved in Aminoglycoside Resistance in *Acinetobacter baumannii* Strain BM4454. *Antimicrob Agents Chemother* **45**, 3375–3380 (2001).

21. S. Coyne, N. Rosenfeld, T. Lambert, P. Courvalin, B. Périchon, Overexpression of Resistance-Nodulation-Cell Division Pump AdeFGH Confers Multidrug Resistance in *Acinetobacter baumannii*. *Antimicrob Agents Chemother* **54**, 4389–4393 (2010).
22. C. K. Stover, *et al.*, Complete genome sequence of Pseudomonas aeruginosa PAO1, an opportunistic pathogen. *Nature* **406**, 959–964 (2000).
23. A. Kumar, K.-L. Chua, H. P. Schweizer, Method for Regulated Expression of Single-Copy Efflux Pump Genes in a Surrogate *Pseudomonas aeruginosa* Strain: Identification of the BpeEF-OprC Chloramphenicol and Trimethoprim Efflux Pump of *Burkholderia pseudomallei* 1026b. *Antimicrob Agents Chemother* **50**, 3460–3463 (2006).
24. S. Sun, A. Negrea, M. Rhen, D. I. Andersson, Genetic Analysis of Colistin Resistance in *Salmonella enterica* Serovar Typhimurium. *Antimicrob Agents Chemother* **53**, 2298–2305 (2009).
25. Y.-H. Cheng, *et al.*, Colistin Resistance Mechanisms in Klebsiella pneumoniae Strains from Taiwan. *Antimicrob Agents Chemother* **59**, 2909–2913 (2015).
